# Low-grade glioma risk SNP rs11706832 is associated with type I interferon response pathway genes in cell lines

**DOI:** 10.1038/s41598-023-33923-4

**Published:** 2023-04-25

**Authors:** Adam Rosenbaum, Anna M. Dahlin, Ulrika Andersson, Benny Björkblom, Wendy Yi-Ying Wu, Håkan Hedman, Carl Wibom, Beatrice Melin

**Affiliations:** 1 Department of Radiation Sciences, Oncology Umeå University, Umeå, Sweden; 2 Department of Chemistry, Umeå University, Umeå, Sweden

**Keywords:** Cancer, Cancer genetics, Cancer genomics, CNS cancer, Cancer genetics, Cancer genomics, CNS cancer, Gene ontology, Genomics, Metabolomics, RNA sequencing, Gene regulation, Haplotypes, RNA splicing

## Abstract

Genome-wide association studies (GWAS) have contributed to our understanding of glioma susceptibility. To date, 25 risk loci for development of any of the glioma subtypes are known. However, GWAS studies reveal little about the molecular processes that lead to increased risk, especially for non-coding single nucleotide polymorphisms (SNP). A particular SNP in intron 2 of *LRIG1*, rs11706832, has been shown to increase the susceptibility for *IDH1* mutated low-grade gliomas (LGG). Leucine-rich repeats and immunoglobulin-like domains protein 1 (LRIG1) is important in cancer development as it negatively regulates the epidermal growth factor receptor (EGFR); however, the mechanism responsible for this particular risk SNP and its potential effect on *LRIG1* are not known. Using CRISPR-CAS9, we edited rs11706832 in HEK293T cells. Four HEK293T clones with the risk allele were compared to four clones with the non-risk allele for *LRIG1* and *SLC25A26* gene expression using RT-qPCR, for global gene expression using RNA-seq, and for metabolites using gas chromatography-mass spectrometry (GC–MS). The experiment did not reveal any significant effect of the SNP on the expression levels or splicing patterns of *LRIG1* or *SLC25A26*. The global gene expression analysis revealed that the risk allele C was associated with upregulation of several mitochondrial genes. Gene enrichment analysis of 74 differentially expressed genes in the genome revealed a significant enrichment of type I interferon response genes, where many genes were downregulated for the risk allele C. Gene expression data of *IDH1* mutated LGGs from the cancer genome atlas (TCGA) revealed a similar under expression of type I interferon genes associated with the risk allele. This study found the expression levels and splicing patterns of *LRIG1* and *SLC25A26* were not affected by the SNP in HEK293T cells. However, the risk allele was associated with a downregulation of genes involved in the innate immune response both in the HEK293T cells and in the LGG data from TCGA.

## Introduction

The WHO classifies gliomas, the most common type of malignant brain tumors, using a scale 1–4^1^. Since 2016, the classification of gliomas has also included *isocitrate dehydrogenase* (*NADP*(+))* 1* and *2* (*IDH1/2*) mutation and 1p19q co-deletion status^2^. High-grade gliomas have a particularly poor prognosis, with grade 4 glioblastoma displaying a five-year survival rate of only 7%^3^. Low-grade gliomas (LGG)–grades I and II–have a considerably better survival rate, in particular grade I pilocytic astrocytomas, which are often curable. Grade II gliomas, however, often progress to high-grade lethal tumors^4^. As available therapies have a modest effect on the survival rate of higher-grade gliomas^5^, improved understanding of the disease etiology could be used to develop future therapies.

Although there are few known environmental factors associated with gliomas, a heritable component for developing glioma at any stage of life has been observed^6,7^. Genome-wide associations studies (GWAS) have helped elucidate common germline variants that increase the susceptibility for the disease. Although GWAS can point to significant loci in the genome, functional studies are needed to understand the molecular mechanisms that lead to disease susceptibility. In a large meta GWAS^8^, we identified several novel glioma risk-associated single nucleotide polymorphisms (SNPs), including rs11706832, which is located in intron 2 of the *leucine-rich repeats and immunoglobulin-like domains protein 1* coding gene *LRIG1* In another study, we found that rs11706832 is associated with the susceptibility for *IDH1* mutated gliomas^9^. LRIG1 is a negative regulator of various receptor tyrosine kinases, including members of the epidermal growth factor receptor family^10^, and a suppressor of platelet-derived growth factor B-driven experimental glioma^11^. Although the location of rs11706832 in intron 2 of *LRIG1* could indicate a role in the regulation of *LRIG1* itself, the SNP is not a documented expression quantitative trait locus (eQTL) for *LRIG1*, but rs11706832 affect the expression of the adjacent *solute carrier family 25 member 26* gene *SLC25A26* according to the GTEx database (https://gtexportal.org/home/snp/rs11706832). It has previously been suggested that the rs11706832 A-C conversion would create a possible perturbation of the binding motif for the transcriptional repressor LEF1^12^^13^. Public data from DNase hypersensitivity sequencing from ENCODE reveals rs11706832 lies in an open chromatin region, indicating a regulatory mechanism of the SNP. In another study SNP rs55705857 (A>G) in *8q24*, also increasing the risk of IDH1-mutated glioma, was shown to disrupt the binding site of OCT2/4 and increase expression of *MYC *^14^. It is reasonable to believe that rs11706832 could affect *LRIG1* or *SLC25A26*, using a similar mechanism. In this study, the effect of rs11706832 allelic variants was studied by modifying the locus in HEK293T cells using CRISPR-CAS9. In addition, we analyzed gene expression using reverse transcription quantitative polymerase chain reaction (RT-qPCR), global gene expression using RNA sequencing (RNA-seq), and metabolites using gas chromatography-mass spectrometry (GC–MS). The potential effect of the SNP on *LRIG1, SLC25A26* and nearby genes as well as global gene expression and metabolites were studied.

## Results

### Generation and characterization of rs11706832-variant HEK293T clones

A modified version of HEK293T cells where one allele of the entire LRIG1 gene has been deleted^15^, was used to study the potential effects of the risk SNP rs11706832 on transcriptomics and metabolomics. Sanger sequencing revealed that the single copy of rs11706832 in the parental cells was the risk *C* allele. rs11706832 of the parental cells was mutated from *C* to the glioma low-risk allele *A* using CRISPR-CAS9*.* Four HEK293T clones with an *A* at the SNP position and four clones with a *C* at the SNP position were isolated. Four replicates of each clone were cultivated independently. The analysis of the 32 cell lines revealed a trend indicating that the cell lines with C allele had a slightly reduced doubling time, although the difference between A and C clones was not significant (p = 0.064) (Fig. S1c).

### Differential gene expression

**Figure 1:**
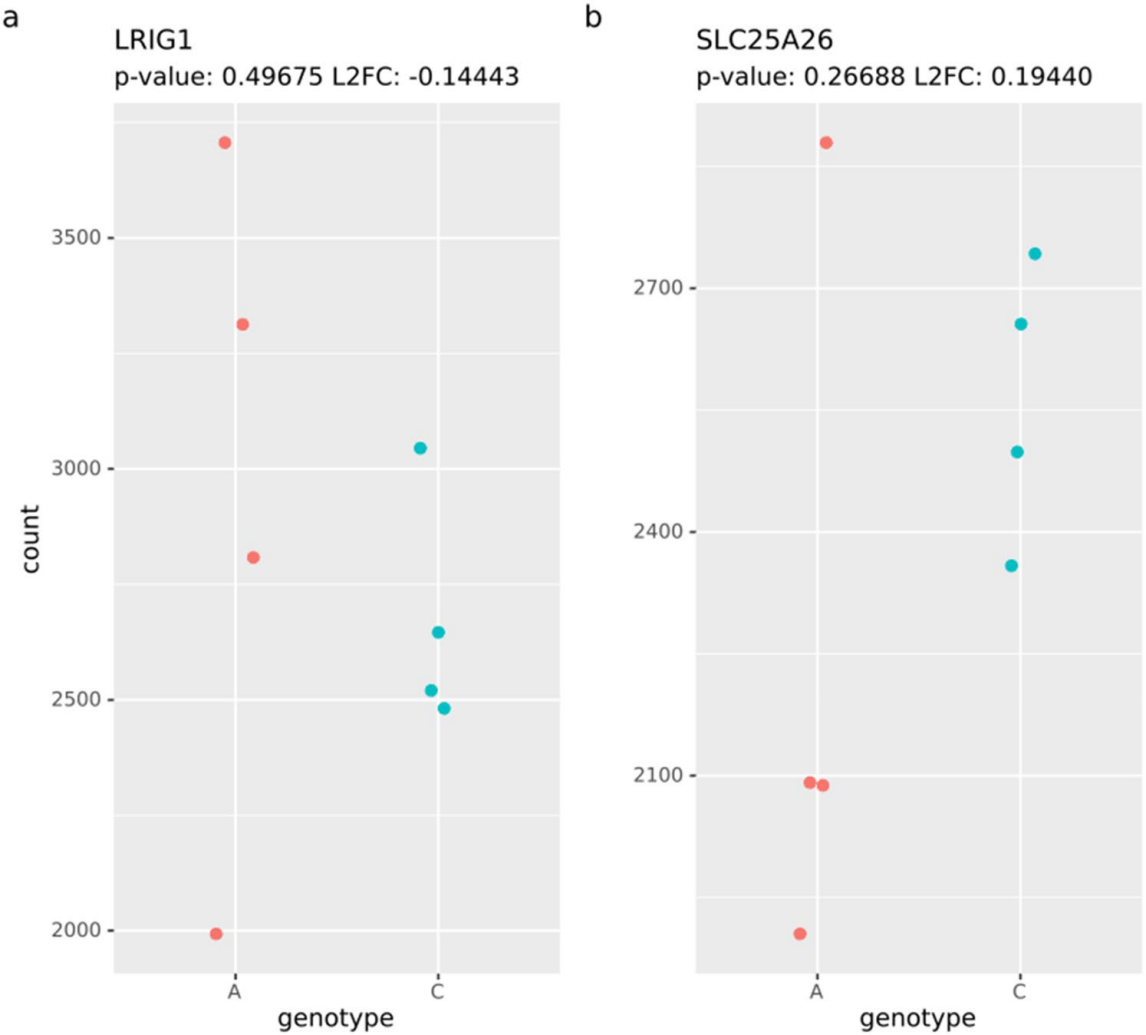
Expression of *LRIG1* and *SLC25A26* in cell lines. Normalized read counts for (**a**) *LRIG1* and (**b**) *SLC25A26*. The low-risk variant (A) to the left and high-risk (C) to the right. Each point is the aggregate for the four replicates.

There was no significant difference in the expression levels of *LRIG1* or *SLC25A26* seen using RT-qPCR (Fig. S1a,b). A region around *LRIG1* of 5 × 10^6^ bases was used to investigate the effects on gene expression near rs11706832 using RNA-seq. Twelve genes were found in this region (Table S1). The only gene having a p-value lower than 0.05 was *MAGI1* (p = 0.025, L2FC = 0.34), showing an increased expression for cell lines with the C risk allele. However, none of the genes were shown to be differentially expressed between cells with different genotypes after correcting for multiple hypothesis testing. Accordingly, neither *LRIG1* nor *SLC25A26* was significantly differentially expressed between the two genotypes as assessed using either RNA-seq (p = 0.50, L2FC =  − 0.14 and p = 0.27, L2FC = 0.19, respectively) (Fig. 1a,b) or RT-qPCR (Fig. S1a,b).

To study any differences in splicing of *LRIG1* and adjacent genes as an effect of rs11706832, we looked at the fraction of reads from the RNA-seq data aligning with specific exons as well as reads aligning over exon junctions. The cell lines showed a similar exon-specific expression regardless of what nucleotide was present on the SNP site (Fig. S3), indicating that the SNP had no effect on splicing *LRIG1* or adjacent genes in the cell lines.

**Figure 2:**
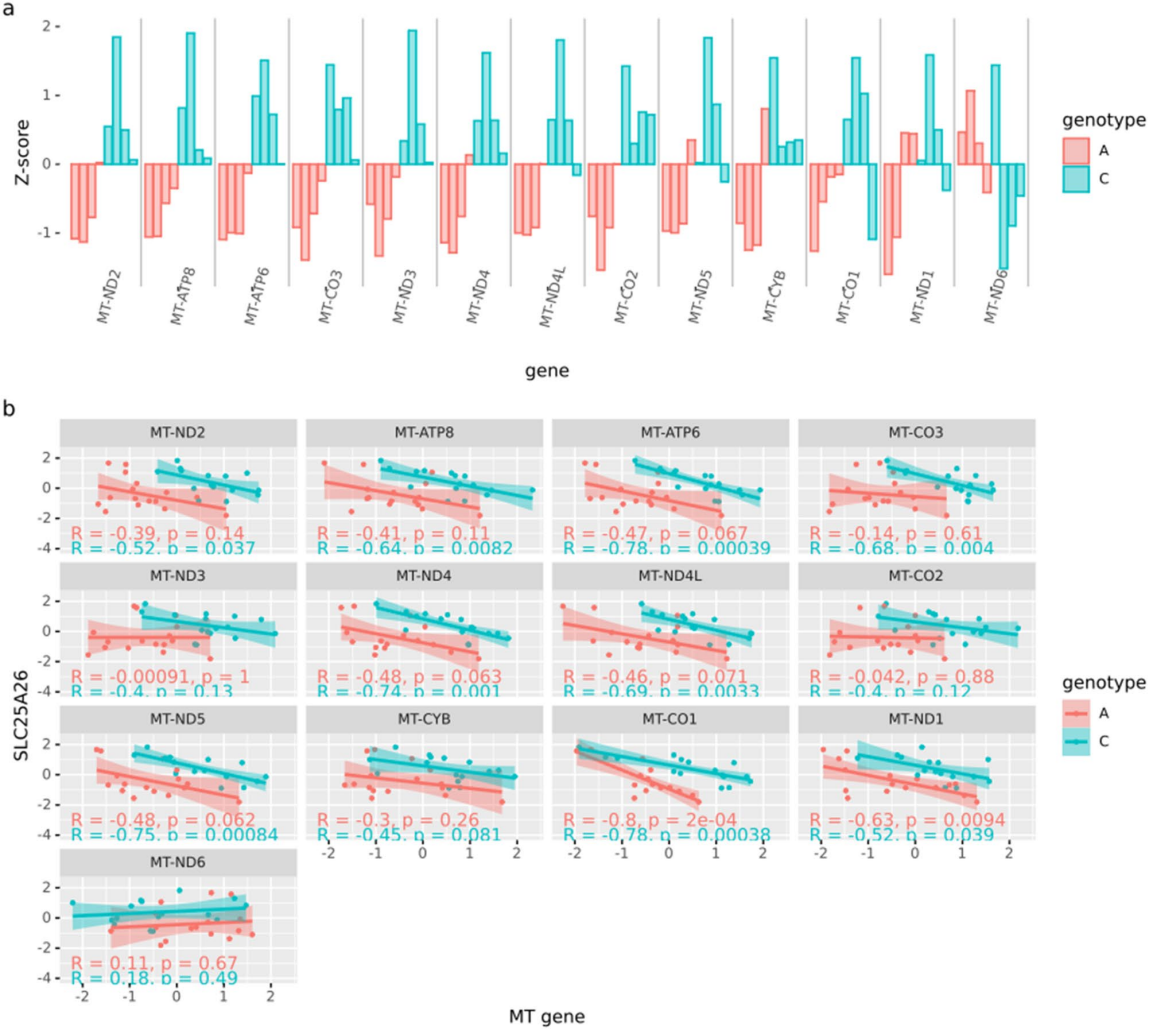
Expression of Mithochondrial genes (**a**) Z-score for expression of 13 mitochondrial genes in cell lines. Cell lines with low-risk nucleotide (A) are red, high-risk genotype (C) blue. (**b**) Correlation between *SLC25A26* and 13 mitochondrial genes for low-risk cell lines (red) and high-risk (blue). Linear correlation and p-values are given in each plot.

SLC25A26 acts a transporter protein of the methyl donor S-adenosylmethionine into the mitochondrion and affects methylation of mitochondrial DNA^16,17^. Therefore, we hypothesized that any alteration of the transcription of *SLC25A26* due to the rs11706832 will affect the transcription of mitochondrial genes even though the upregulation of the genes was small. To investigate if rs11706832 affects the transcription of the mitochondrial genome, all 13 genes on mitochondrial DNA detected in our data were tested for differential expression (Fig. 2a, Table S3). Twelve of the genes showed a statistically significant increased expression in cell lines with the C LGG risk allele. We used principal component analysis (PCA) to detect the general variance of mitochondrial expression of the cell lines. The cell lines were well separated by the first principal component (p = 0.01627 two-sided t-test), including all 13 mitochondrial genes, a finding that suggests a general mitochondrial overexpression for the C risk allele. For most mitochondrial genes, a reverse association between the expression of *SLC25A26* and the mitochondrial gene was observed. This relationship was generally stronger in cells with the C allele (Fig. 2b).

Our global gene expression analysis revealed 74 differentially expressed genes for the A and C allele after FDR correction for all 15,919 tested genes. (Fig. S2, Table S2). For the 74 genes, 14 GO terms were significantly enriched for (adjusted p-value < 0.05). we observed a significant enrichment for several Gene Ontology (GO) terms related to the innate immune system (Table S4). The most significant hit was cellular response to type I interferon (GO:0071357) with the genes *IFITM1*, *SP100*, *OAS1*, *IFI35*, *IFIT3*, *IFIT5*, and *IFIT2* being differentially expressed in the HEK293T cells. For the term innate immune response (GO:0045087) *TRIM17*, *TLR3*, *OAS1*, *CXCL16*, *SP100*, *TRIM34*, *TRIM14*, *B2M*, *IFITM1* were differentially expressed in the HEK293T cells. One term not related to the innate immune system, negative regulation of cell population proliferation (GO:0008285) with genes *NMI*, *PTPN6*, *IFIT3*, *IFI35*, *B2M*, *PARP10*, *IFITM1*, differentially expressed was also significantly enriched for.

### TCGA LGG samples

**Figure 3:**
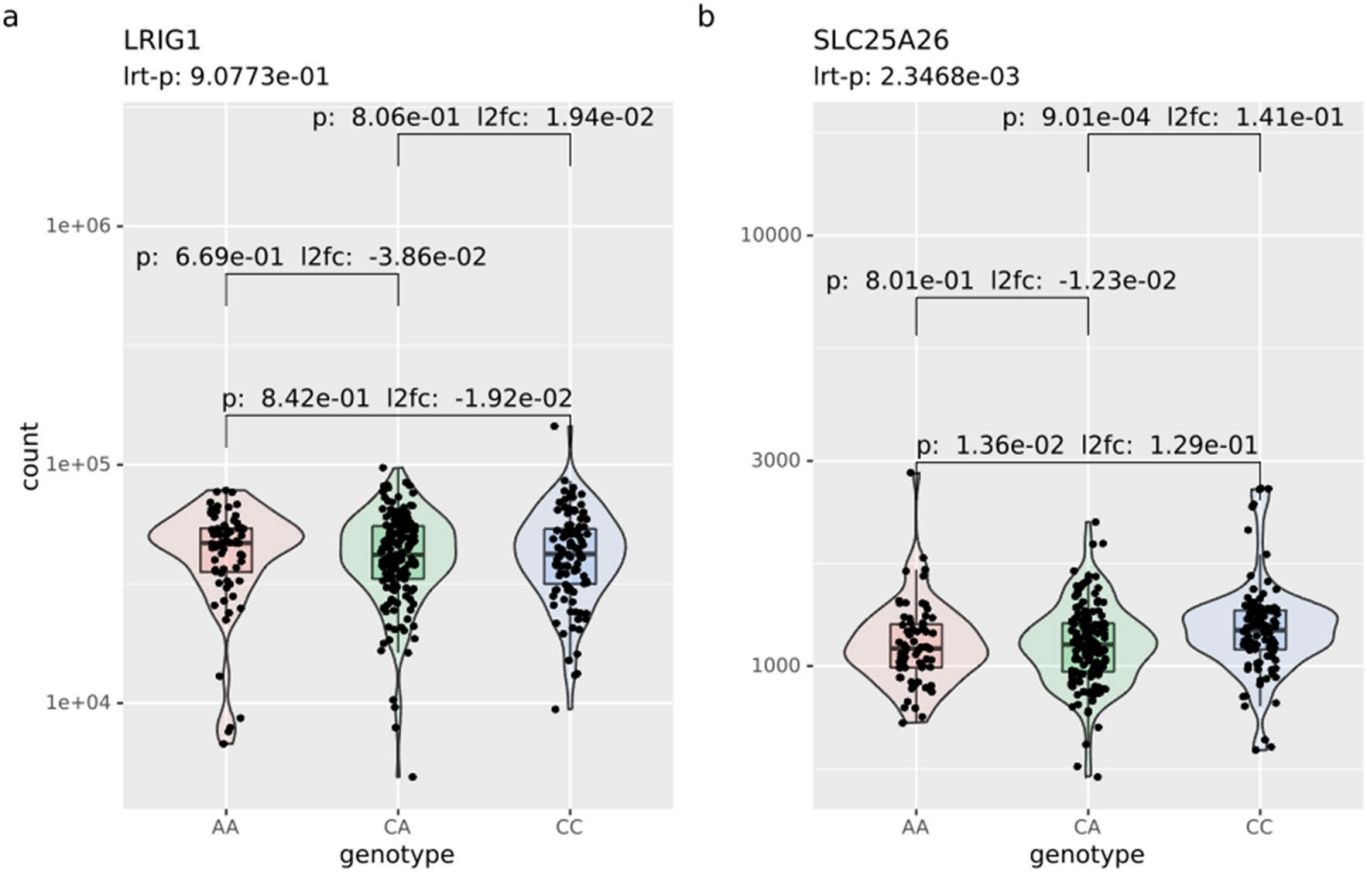
Expression of *LRIG1* and *SLC25A26* in TCGA LGG tumors. Normalized gene counts for (**a**) *LRIG1* and (**b**) *SLC25A26* from TCGA data for IDH-mutated low-grade gliomas. p-values using the Wald test and log-twofold count between all genotypes are shown. p-values from likelihood ratio testing across all genotypes are shown above plot.

**Figure 4:**
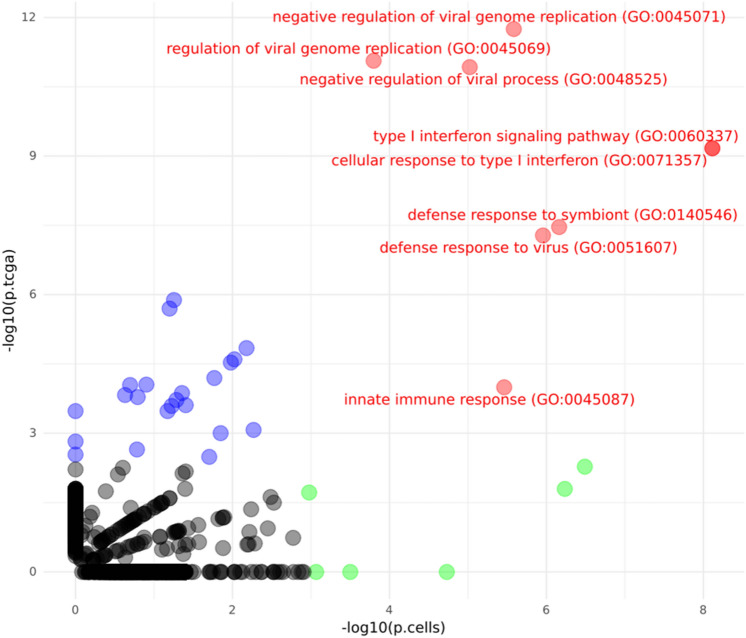
Enriched GO Pathway Terms in Cell Lines vs. TCGA data. -log_10_ p-values from hypergeometric test from enrichment analysis for differentially expressed genes in cell lines (x-axis) and TCGA (y-axis). Red color indicates gene sets where adjusted p < 0.05 in both cell lines and TCGA enrichment analysis, blue only TCGA, and green only cell lines.

As rs11706832 has a specific susceptibility for *IDH1*-mutated low-grade gliomas, we validated the genes found in the HEK293T cell lines using gene count data from TCGA for 325 *IDH1* mutated primary LGGs^9^. Tumors homozygous for the risk allele (C/C, n = 104) had a higher expression of *SLC25A26* compared to tumors homozygous for the non-risk allele (A/A, n = 68) (p = 0.014, L2FC = 0.13), a difference that was also seen between the A/C (n = 153) and C/C samples (p = 9.01e−4, L2FC = 0.14) (Fig. 3b). There was an overall significant difference of expression for the gene after using a likelihood-ratio test (P-LRT = 2.35e−3). No significant difference in *LRIG1* could be observed with likelihood-ratio testing or with pairwise comparisons between the possible genotypes (Fig. 3a). A global analysis revealed that 40 differentially expressed genes in the TCGA data based on likelihood-ratio testing (adjusted p-value < 0.05) (Fig. S4, Table S5). Among these genes, *OAS1* were also identified as significantly expressed in the global analysis of the cell lines. Gene set enrichment of all 40 genes largely overlap the significant terms found in the cell lines, with eight terms all related to the innate immune response, with an adjusted p < 0.05 in both the cell lines and the TCGA data (Fig. 4). For example, the cellular responses to type I interferon (GO:0071357), with an adjusted p = 2e−7 (Table S7), were significantly enriched in the TCGA data as well but with genes *RSAD2*, *OAS1*, *OAS3*, *MX1*, *ISG15*, and *IFIT1.* We investigated the expression for 53 genes in the same Gene Ontology term that was found in the cell lines (cellular response to type I interferon GO:0071357) in the TCGA data; 18 of the 53 genes had a likelihood ratio p-value < 0.05, and 13 genes had a likelihood ratio p-value < 0.05 after BH correction (Table S8). As in the cell lines, these genes were under expressed in the LGG glioma risk genotype C/C compared to the A/A genotype. The reverse association found in the cell lines between *SLC25A26* and the mitochondrial genes were not as prevalent as in the TCGA data, and no over expression of mitochondrial genes for the C/C genotype was observed (Table S6).

### Metabolites

To investigate whether rs11706832 affected metabolic programs, we quantified soluble intracellular metabolite levels using GC–MS for the A or C modified cell lines. Assessing a total of 111 metabolites (Table S9), we found no significant metabolites after univariate statistics and correction for multiple testing at FDR 0.05. Suspecting a high degree of correlated variables, we used an unsupervised PCA for the 111 quantified metabolites to look for a general pattern of variance between the genotypes. Genotypes were separated along the first and third principal components (Table S10), indicating a difference in metabolite levels between the A and C allele, although the over-all modeled effect was small (PC1: p = 0.073, PC3: 0.090, two-sided t-test). To investigate what metabolites contributed to the separation, we considered each cell line replicate as an independent measure and repeated the two-sided t-test. Both 2-aminoadipic acid (p = 0.00045, FC = 1.99) and sphingosine (p = 0.0019, FC = 1.59) (Fig. S5a,b) were found at a significantly higher concentrations for the A allele. Interestingly, both metabolites have previously been shown to be metabolic hallmarks of high-grade glioma and exist in high concentrations in high-grade glioma tumor tissue compared to LGG tumors^18^.

## Discussion

The glioma risk SNP rs11706832 is located in intron 2 of *LRIG1*, but this study found no apparent effect on *LRIG1* expression level in the genetically modified HEK293T cell lines. This result was consistent in both RNA-seq and RT-qPCR data. Furthermore, the RNA-seq data did not reveal any obvious effect on splicing as a result of the SNP in the cell lines. These results together with public data from GTEx, and TCGA, suggests that the SNP does not affect the transcription of *LRIG1* and that differential expression of this gene does not explain the increased risk of glioma development conferred by rs11706832. The expression of *SLC25A26* has an effect in the same direction as seen in GTEx and TCGA for the high-risk C allele. This upregulation is however non-significant both in RT-qPCR and RNA-seq.

The investigation of the downstream effects that an overexpression of *SLC25A26* would confer on the cell lines showed a difference between the A and C cell lines in the expression pattern of several mitochondrial genes. Expression of several mitochondrial genes was negatively correlated to expression of *SLC25A26*, and this correlation was stronger for the cell lines with the risk allele than for the cell lines with the non-risk allele. The reverse association between mitochondrial gene expression and *SLC25A26* expression is in line with the involvement of *SLC25A26* in methylation of mitochondrial DNA. We found an indication that mitochondrial gene expression was upregulated in cell lines having the risk allele C. However, these findings could not be validated in the TCGA data.

Using RNA-seq data from the cell lines, we identified several genes involved in the interferon response and innate immune response to be downregulated in cell lines with the risk allele C. Among these are *IFIT2*, *IFIT3*, and *IFIT5* (coding for interferon induced proteins with tetratricopeptide repeats 2, 3, and 5) primarily induced by interferons of type I, and plays part in the response to viral infections^19^. The 2’-5’-oligoadenylate synthetase 1 protein coded by *OAS1*, also have an important antiviral role and acts by activating RNase L^20^. Three genes coding for tripartite motif-containing proteins *TRIM17*, *TRIM34*, *TRIM14* were also differentially expressed, as was the toll-like receptor 3 coding gene *TLR3*. TLR3 has been shown to induce type I interferons^21^, and several TRIM proteins are induced by type I interferons^22^. Some of these genes has also been implicated various cancers^23–25^. Although *OAS1* was the only globally differentially expressed gene in TCGA, enrichment for the same GO term of type I interferon response and innate immune response as in the cell line. Additional analysis in TCGA revealed 13 differentially expressed genes in this gene set that were significantly downregulated for the risk allele. Although a downregulation of interferon response could give an explanation of an increased risk of developing glioma, there is currently no mechanism explaining how the glioma risk SNP rs11706832 is involved in the downregulation of type I interferon response genes. One potential mechanism could be through altered mitochondrial gene expression of genes involved in oxidative phosphorylation due to a regulatory effect of *SLC25A26* by the SNP. The mitochondria has been implicated in the innate immune response through several mechanisms^26^. Notably, although no alteration of *LRIG1* expression was seen in this study, high expression of *LRIG1* has been associated with an interferon response signature in primed quiescent neural stem cells^27^.

In the cell lines, genes in the negative regulation of cell population proliferation GO term were differentially expressed. Such genes, like the *N-myc-interactor NMI* gene differentially expressed in the cell lines, may seem highly interesting and have been shown relevant to several cancer types^28^. Furthermore, *NMI* has been shown to be interferon induced^29^. However, these genes were not found differentially expressed in the TCGA data.

Our analysis of the concentrations of 111 metabolites in the cell lines through GC–MS showed no significant difference in metabolite concentration between the A and C cell lines after multiple hypothesis testing, and it is not possible to say anything conclusive on the effect rs11706832 has on metabolism in the cell lines.

One should also consider possible off-target effects from the CRISPR-CAS9 procedure and whether this could confound the results. As all parental cells were subjected to the same CRISPR-CAS9 procedure, any off-target effect would likely be present randomly in the cell lines regardless of the resulting rs11706832 genotype. However, in principle, it is also possible that the reason why certain cells acquired the A nucleotide at rs11706832 and other cells did not could be due to inherent differences between the respective cell types. This possibility cannot formally be excluded. However, because a similar phenotypical difference with regards to the innate immune response genes was observed between the genotypes in the cell lines and in the TCGA LGG tumor data, we favor the interpretation that the phenotypical difference between the cell lines is due to rs11706832 rather than to off-target effects*.*

A limitation of this study was the use of a non-glial cell line. We chose to use the human embryonic kidney cell line HEK293T to analyze the cellular effects of the two SNP variants because we had access to a HEK293T subclone that was haploid at the *LRIG1* locus, which greatly simplified the genetic modifications and analyses of the SNP. However, it is reasonable to believe that effects from risk predisposition SNPs are to some degree cell type specific, and that other effects might have been observed if we had used a glial cell line. This problem is further complicated by our limited knowledge about the identity of the cellular origin of low-grade glioma and at what stage during tumorigenesis the risk SNP plays its role.

In conclusion, the data in this and previous studies suggest that the increased risk of *IDH1*-mutated glioma conferred by rs11706832 is not caused by an effect on the *LRIG1* expression level*.* Instead, the most notable effect conferred by the rs11706832 alleles in the cell lines was the downregulation of genes involved in the innate immune response and interferon response, which was also observed in the TCGA LGG data. However, the mechanistic link between rs11706832 and the immune and interferon responses remains to be elucidated.

## Methods

### Modifying HEK293T Cell lines

The HEK293T cell line with only one copy of *LRIG1* was generated through CRISPR-Cas9-mediated ablation of one copy of the entire gene as has previously been described^15^. Sanger sequencing revealed that the *LRIG1* single-allelic HEK293T cell line carried the C allele at rs11706832. To introduce the A allele, the cells were transiently co-transfected with plasmids carrying the gRNA 5’-*GTACTTGCGATGCACAGTCA-3’,* Cas9*,* and the single-stranded oligodeoxynucleotide 5’-aaactgagaataagaacccagttttatccccttgact**T**tgcatcgcaagtacaatttctttctagcccatcacctggcagaaagcctgaacactttgctttggttttcttaaggattgttgggctca-3’ (the modified site indicated with a capital bold T). Thereafter, the transfected cells were cloned and their rs11706832 variants were determined through Sanger sequencing. Four successful knock-in clones (A allele; T2-1, T2-10, T2-14, and T2-44) and four negative knock-in clones (C allele; T2-2, T2-19, T2-24, and T2-28) were isolated and used in the study. The whole CRISPR-Cas9 procedure was performed by GenScript USA Inc (Piscataway, NJ, USA). Four replicate cell lines of each clone were cultivated separately in Dulbecco’s modified Eagle’s medium with 10% fetal bovine serum and 50 µg/mL gentamicin. Cells were cultivated for 11 days. At day 11, cells were collected for metabolomics analyses and isolation of mRNA were purified for RT-qPCR. The remaining cells were frozen at − 80 °C and sent for RNA-seq.

### RNA-extraction and quantitative RT-PCR-analyses

For qRT-PCR and RNA sequencing, RNA was prepared using a Dynabeads mRNA Direct Kit (Ambion, Fisher Scientific GTF AB, Gothenburg, Sweden) according to the manufacturer’s instructions. The TaqMan gene expression assays for LRIG1 exon junction 2–3 (Hs01006146_m1), *LRIG1* exon junction 10–11 (Hs01006138_m1), and *SLC25A26* (Hs01115565_m1) were purchased from Fisher Scientific GTF AB. Primers and probes for the housekeeping genes *GJB2*, *CHUK*, and *HTR2C* have been previously described^30^. Data were acquired using a CFX96 system C1000 thermal cycler (Bio-Rad Laboratories AB) as previously described^31^. The specific gene expression levels were normalized to that of the houskeeping genes by transforming the ΔCT values from log2 to linear values.

### RNA library preparation and sequencing

RNA sequencing of the cell lines was performed by the SNP&SEQ Technology Platform (Uppsala, Sweden). Sequencing libraries for all 32 samples were prepared from 100 ng polyA selected RNA using the TruSeq stranded mRNA library preparation kit (Cat# 20020594/5) without the polyA selection step. The library preparation was performed according to the manufacturer’s protocol (#1000000040498). Sequencing was carried out using NovaSeq S4 flow cell to paired-end 150 bp reads with v1 sequencing chemistry. This resulted in ~ 60 M read pairs per sample.

### Gas chromatography mass spectroscopy

Soluble intracellular metabolites were extracted from snap frozen cell pellets containing 4 million cells. Metabolites were extracted on ice at 4 °C and with an 18-mg/ml methanol:water extraction mix (90:10 v/v) including labeled internal standards (2.5 ng/μl, myristic acid-13C3, cholesterol-D7 (Cil, Andover, MA, USA) and D-sucrose-13C12 (Sigma-Aldrich, St. Louis, MO, USA). Samples were homogenized using rigorous agitation at 30 Hz for 2 min in a bead mill (Retsch, MM 400) followed by protein precipitation at − 20 °C for 2 h and centrifugation at 18,600×*g* for 10 min at 4 °C. A 200-μl extract was transferred to glass vials and evaporated until dry in a SpeedVac. Samples containing extracted metabolites from eight cell lines carrying either the A or C genotype were randomized within four analytical batches, each batch containing technical replicates harvested on four different days for a total of 32 samples. Pooled quality control (QC) reference samples were included at the beginning and end of each analytical batch and after every eight analytical samples for monitoring of platform performance. Dried samples were dissolved in pyridinic methoxyamine, derivatized, and analyzed by GC-EI-MS as described by us^32^. The identities of the resolved peaks were determined by comparing mass spectra and retention indices with data in the Swedish Metabolomics Centre in-house spectral library. NIST MS search 2.3 software was used for manual verification of spectral identification. For identification with high confidence, we ensured all major fragment ions in the library hit were present in the resolved spectra with correct spectral intensity profile and retention index. The median RSD_QC_% for all identified metabolites was 7.5%, and 94.3% of the identified metabolites had an RSD_QC_% below 30%. The median NIST match identification score value was 887 and median deviation in Kovats retention index was 4.0.

### Preprocessing of RNA-seq samples

After adapter-trimming with *trim-galore* (v. 0.4.4), paired-reads were quantified against the *Ensembl* total cDNA (v. 99) using the *salmon* (v. 0.14.1)^33^. The *salmon* quantification data were used in the differentially expression analysis. A full alignment of the paired reads was made using the *STAR* (2.7.3)^34^ against the *Ensembl* reference genome GRCh38 release 99, followed by a guided transcript assembly based on the *Ensembl* gene annotation release 99 The exon and intron-junction count from *stringtie* (v. 2.1.2)^35^ were used in the differential splicing analysis.

### Differential expression analysis

The transcript quantification produced from *salmon* was previously aggregated to the gene level for further analysis with the *R* package *tximport* (v. 1.14.0). Differential gene expression was analyzed with the *R* package *DESeq2* (v. 1.26.0)^36^. Genes with fewer than 10 mapped reads for more than half of the samples were filtered from downstream analysis. Replicates for all cell lines were aggregated before testing for differential expression. A targeted analysis for genes within 10^6^ bases of LRIG1 that fulfilled the filtering criteria were tested for differential expression between the genotypes. A global analysis for all genes fulfilling the criteria were tested, correcting for multiple hypothesis using the Benjamini-Hochberg (BH) method. All genes with an adjusted p < 0.05 were considered as differentially expressed and a Log_2_ Fold-change (L2FC) > 0.5.

Raw gene count data for IDH1 mutated primary low-grade gliomas from TCGA were downloaded. Samples where the genotype for the rs11706832 were known were used for differential expression analysis with DEseq2 with the same filtering criteria as for the cell lines. Pairwise difference in expression between all three genotypes (A/A, A/C, and C/C) was tested with Wald’s test. Differential expression across all three genotypes were made using likelihood-ratio tests (LRT). Genes with an adjusted p < 0.05 based on the LRT were considered differentially expressed in the global analysis.

### Gene set enrichment analysis

Gene set enrichment analysis was carried out with the Bioconductor *clusterProfiler* (v 3.15.0)^37^ R package against the gene set GO_Biological_Processes_2021^38^. Hypergeometric tests were performed using the list of significantly expressed genes against the GO gene set.

### Differential splicing

Reads mapping to specific exons and exon-exon junctions were quantified using *stringtie*. For all genes with more than one exon within 10^6^ bases within *LRIG1*, expression of a specific exon was calculated as the fraction of mapped reads to the specific exon compared to the total reads mapping to any exon in the same gene. The fractions of reads spanning exon junctions were calculated in the same way. Exons or exon junctions with a fraction of < 0.01 were filtered. These fractions were used to assess potential differences in exon use or differential splicing between the cell lines.

### Analysis of metabolites

The mean metabolite intensity from GS-MS was obtained from all four replicates of each cell line. Z-score was calculated for each metabolite, and univariate testing with two-sided t-test was made for each metabolite. This method was done by treating the replicates for each cell lines as technical replicates for aggregating the replicate data and by treating each replicate as an independent measurement. The BH method was used to adjust p-values from the tests. A metabolite was considered significant if the adjusted p-value was less than 0.05 and L2FC was greater than 0.5. PCA was performed with the *PCAtools* (v. 1.2.0) package in R.

## Supplementary Information

Supplementary Information 1.

Supplementary Information 2.

Supplementary Information 3.

Supplementary Information 4.

Supplementary Information 5.

Supplementary Figures.

Supplementary Table S1.

Supplementary Table S2.

Supplementary Table S3.

Supplementary Table S4.

Supplementary Table S5.

Supplementary Table S6.

Supplementary Table S7.

Supplementary Table S8.

Supplementary Table S9.

Supplementary Table S10.

## Data Availability

RNA-seq fastq-files are available at SRA with accession number PRJNA846525. Cell proliferation, qRT-PCR, and GC–MS data are attached as Supplementary Information 2–5 in the supplementary materials. Open access gene count data and clinical data for TCGA LGG tumor samples was downloaded through National Cancer Institute, GDC Data Portal https://portal.gdc.cancer.gov/. Manifest file for downloaded files attached as supplementary materials. Controlled access germline genotype data for TCGA individuals were downloaded from the GDC Legacy Archive.
