# Supplementary material for: Low-grade glioma risk SNP rs11706832 is associated with type I interferon response pathway genes in cell lines
Source: Sci Rep. 2023 Apr 25;13:6777. doi: 10.1038/s41598-023-33923-4 (PMC10130147; doi:10.1038/s41598-023-33923-4)
Supplement: Supplementary file 6 — Supplementary Figures. [file 41598_2023_33923_MOESM6_ESM.docx]

## Figure S1. RT-qPCR and Proliferation in Cell Lines.


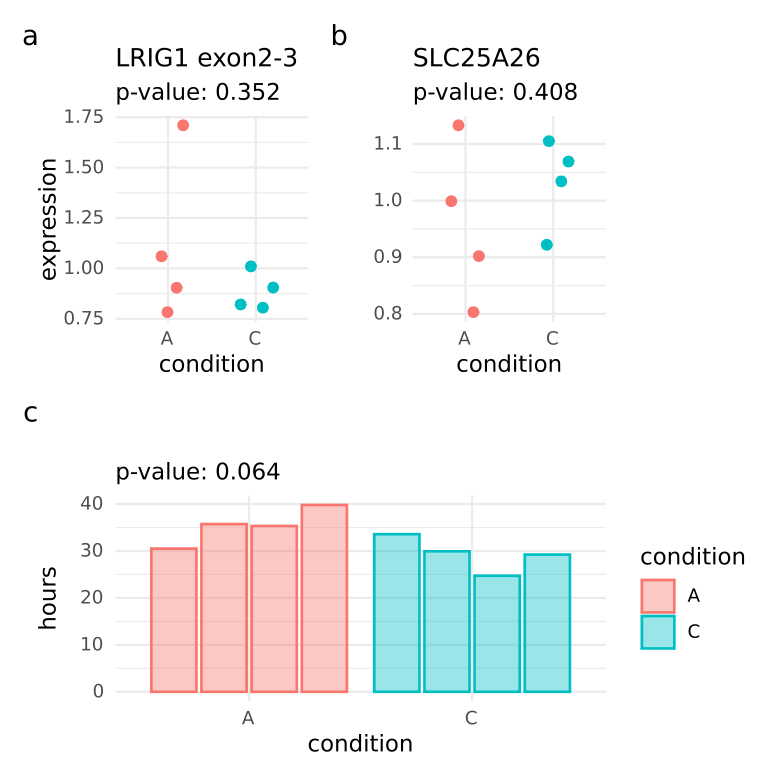


**a), b)** Gene expression from RT-qPCR for LRIG1 and SLC25A26. **c)** Doubling time for cells with either genotype.

## Figure S2. Differentially Expressed genes in Cell lines.


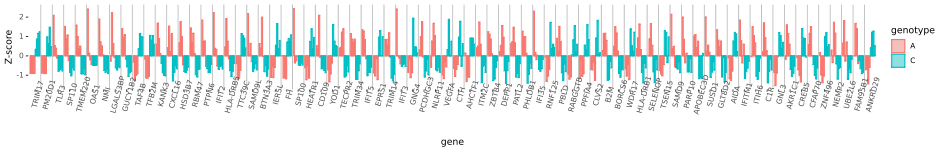


Z-Score for all 74 significant differentially expressed genes in cell lines between A and C genotype. Ordered by p-value (lower to higher).

## Figure S3. Splicing of *LRIG1* and *SLC25A26* in Cell Lines.


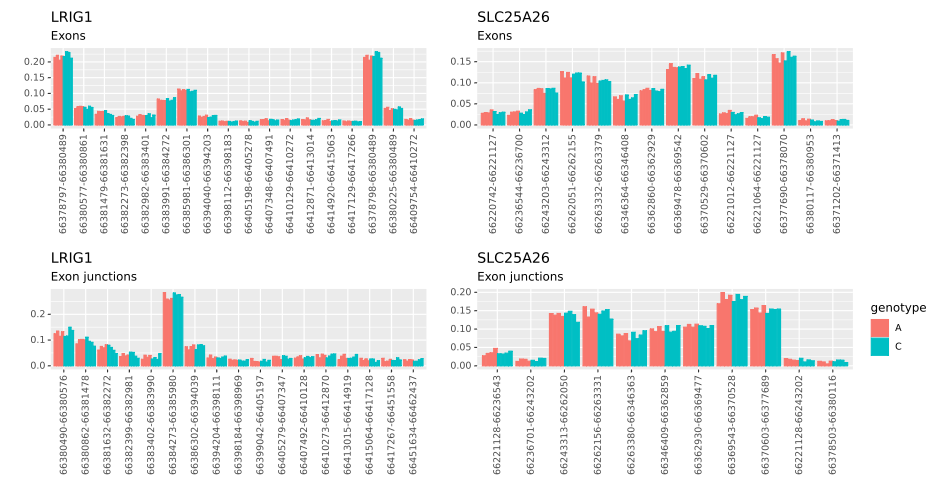


Exon (above) and exon-junction (below) proportions for SLC25A26 and LRIG1. Bars are colored by low-risk (A) and risk (C) genotypes.

## Figure S4. Differentially Expressed genes in TCGA LGG Tumor Samples.


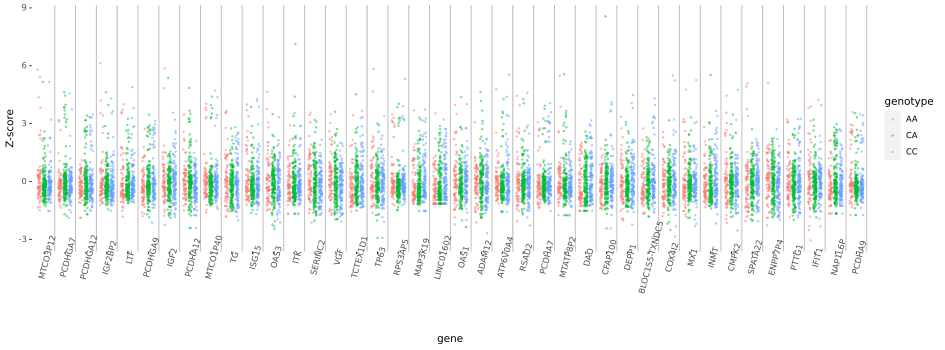


Z-score for 40 significant differentially expressed genes in TCGA IDH1-mutated low-grade gliomas. Ordered by p-value (lower to higher).

## Figure S5. Concentration of Metabolites in Cell Lines.


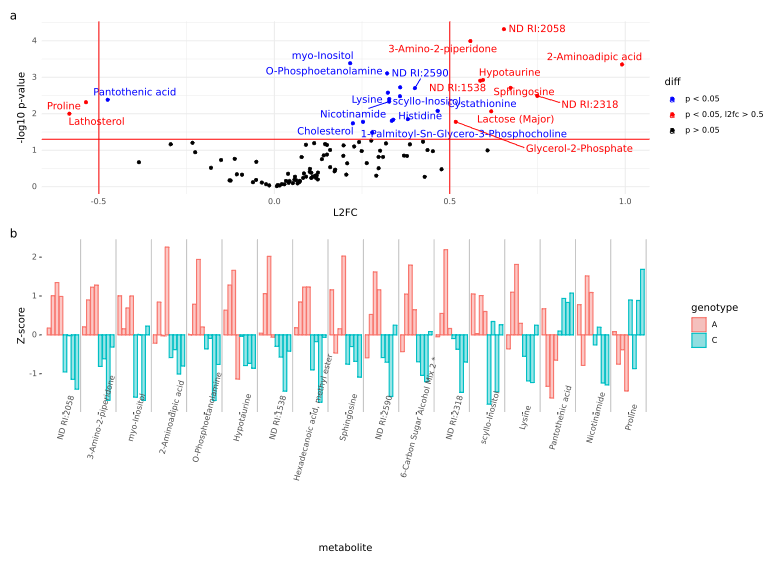


**a)** Volcano plot of metabolites. Horizontal line at p = 0.05 and vertical lines at −0.5 and 0.5 **b)** Z-score for metabolites with different concentration between genotypes. Ordered by p-value (lower to higher).
