# Supplementary material for: Low-grade glioma risk SNP rs11706832 is associated with type I interferon response pathway genes in cell lines
Source: Sci Rep. 2023 Apr 25;13:6777. doi: 10.1038/s41598-023-33923-4 (PMC10130147; doi:10.1038/s41598-023-33923-4)
Supplement: Supplementary file 7 — Supplementary Table S1. [file 41598_2023_33923_MOESM7_ESM.docx]

|  | baseMean | log2FoldChange | lfcSE | stat | Pvalue | padj | gene_name |
| --- | --- | --- | --- | --- | --- | --- | --- |
| ENSG00000151276 | 9432.5479 | 0.3381104 | 0.1508968 | 2.2406727 | 0.0250473 | 0.3005674 | *MAGI1* |
| ENSG00000163636 | 26974.3597 | 0.2453493 | 0.2127654 | 1.1531443 | 0.2488511 | 0.6864535 | *PSMD6* |
| ENSG00000163638 | 3708.9820 | 0.2076792 | 0.1946588 | 1.0668882 | 0.2860223 | 0.6864535 | *ADAMTS9* |
| ENSG00000144741 | 2401.8460 | 0.1943997 | 0.1750915 | 1.1102747 | 0.2668807 | 0.6864535 | *SLC25A26* |
| ENSG00000163376 | 5445.3242 | -0.2503511 | 0.1913861 | -1.3080944 | 0.1908413 | 0.6864535 | *KBTBD8* |
| ENSG00000144749 | 2813.6713 | -0.1444343 | 0.2125269 | -0.6796048 | 0.4967547 | 0.8515796 | *LRIG1* |
| ENSG00000163378 | 8609.0124 | -0.2080418 | 0.2765136 | -0.7523747 | 0.4518257 | 0.8515796 | *EOGT* |
| ENSG00000172340 | 11919.5270 | -0.0994308 | 0.2120638 | -0.4688719 | 0.6391612 | 0.8522150 | *SUCLG2* |
| ENSG00000163377 | 149.8202 | -0.2324586 | 0.4295243 | -0.5412000 | 0.5883697 | 0.8522150 | *TAFA4* |
| ENSG00000163635 | 11188.3052 | 0.0549936 | 0.2016970 | 0.2726542 | 0.7851190 | 0.8939725 | *ATXN7* |
| ENSG00000163637 | 1539.3079 | 0.0325312 | 0.2196344 | 0.1481152 | 0.8822519 | 0.8939725 | *PRICKLE2* |
| ENSG00000241506 | 211.1749 | -0.0577936 | 0.4336274 | -0.1332793 | 0.8939725 | 0.8939725 | *PSMC1P1* |

# S1. Differential expression of genes 2.5Mb around *LRIG1 in cell lines C vs. A at SNP position*

**baseMean**

mean normalized count across all samples

**log2FoldChange**

log_2_ fold change

**lfcSE**

standard error of log_2_ fold change

**stat**

Wald statistic

**Pvalue**

p-value from Wald test

**padj**

Benjamini-Hochberg corrected p-value

Sorted on **Pvalue**
