# Supplementary material for: Low-grade glioma risk SNP rs11706832 is associated with type I interferon response pathway genes in cell lines
Source: Sci Rep. 2023 Apr 25;13:6777. doi: 10.1038/s41598-023-33923-4 (PMC10130147; doi:10.1038/s41598-023-33923-4)
Supplement: Supplementary file 8 — Supplementary Table S2. [file 41598_2023_33923_MOESM8_ESM.docx]

# S2. Differential expressed genes in cell lines C vs. A at SNP position

**baseMean**

mean normalized count across all samples

**log2FoldChange**

log_2_ fold change

**lfcSE**

standard error of log_2_ fold change

**stat**

Wald statistic

**Pvalue**

p-value from Wald test

**padj**

Benjamini-Hochberg corrected p-value

Sorted on **Pvalue**

|  | baseMean | log2FoldChange | lfcSE | stat | pvalue | padj | gene_name | chrom | start | end |
| --- | --- | --- | --- | --- | --- | --- | --- | --- | --- | --- |
| ENSG00000162931 | 577.6836 | 6.6226423 | 0.4236525 | 15.632251 | 0.0000000 | 0.0000000 | *TRIM17* | chr1 | 228407935 | 228416861 |
| ENSG00000162877 | 165.1789 | 6.1353411 | 0.5849944 | 10.487863 | 0.0000000 | 0.0000000 | *PM20D1* | chr1 | 205828025 | 205850132 |
| ENSG00000164342 | 1097.2302 | -2.5117952 | 0.3634521 | -6.910938 | 0.0000000 | 0.0000000 | *TLR3* | chr4 | 186069155 | 186088073 |
| ENSG00000135899 | 3161.5008 | -2.1844472 | 0.3511310 | -6.221174 | 0.0000000 | 0.0000015 | *SP110* | chr2 | 230167293 | 230225729 |
| ENSG00000187824 | 697.9411 | -1.4134381 | 0.2262623 | -6.246901 | 0.0000000 | 0.0000015 | *TMEM220* | chr17 | 10699015 | 10730023 |
| ENSG00000089127 | 421.2455 | -4.7931344 | 0.7752949 | -6.182337 | 0.0000000 | 0.0000016 | *OAS1* | chr12 | 112906783 | 112933222 |
| ENSG00000123609 | 1207.8433 | -1.2893903 | 0.2371260 | -5.437576 | 0.0000001 | 0.0001202 | *NMI* | chr2 | 151270470 | 151289894 |
| ENSG00000108679 | 25668.4387 | -1.9923395 | 0.3737368 | -5.330862 | 0.0000001 | 0.0001904 | *LGALS3BP* | chr17 | 78971238 | 78979947 |
| ENSG00000123201 | 1565.7480 | -1.0946018 | 0.2228282 | -4.912314 | 0.0000009 | 0.0015584 | *GUCY1B2* | chr13 | 50994511 | 51080862 |
| ENSG00000141384 | 11334.0972 | 0.6872060 | 0.1428883 | 4.809393 | 0.0000015 | 0.0023591 | *TAF4B* | chr18 | 26226445 | 26391685 |
| ENSG00000162851 | 2673.9891 | 0.8362320 | 0.1792890 | 4.664157 | 0.0000031 | 0.0041147 | *TFB2M* | chr1 | 246540561 | 246566261 |
| ENSG00000186994 | 897.6020 | -1.1708701 | 0.2512826 | -4.659574 | 0.0000032 | 0.0041147 | *KANK3* | chr19 | 8322584 | 8343262 |
| ENSG00000161921 | 8856.6973 | -0.8436723 | 0.1822073 | -4.630289 | 0.0000037 | 0.0043771 | *CXCL16* | chr17 | 4733531 | 4739928 |
| ENSG00000099377 | 1352.4506 | -1.3908339 | 0.3027912 | -4.593377 | 0.0000044 | 0.0045889 | *HSD3B7* | chr16 | 30985207 | 30989147 |
| ENSG00000163694 | 2191.7249 | -0.7246347 | 0.1578477 | -4.590721 | 0.0000044 | 0.0045889 | *RBM47* | chr4 | 40423267 | 40630875 |
| ENSG00000111679 | 1666.0961 | -1.6790618 | 0.3785261 | -4.435789 | 0.0000092 | 0.0089345 | *PTPN6* | chr12 | 6946468 | 6961316 |
| ENSG00000119922 | 550.3710 | -2.3021657 | 0.5226334 | -4.404934 | 0.0000106 | 0.0096996 | *IFIT2* | chr10 | 89283694 | 89309271 |
| ENSG00000198502 | 714.8064 | -2.0293334 | 0.4622535 | -4.390088 | 0.0000113 | 0.0098091 | *HLA-DRB5* | chr6 | 32517353 | 32530287 |
| ENSG00000168234 | 4680.1927 | 0.5749227 | 0.1317785 | 4.362794 | 0.0000128 | 0.0100052 | *TTC39C* | chr18 | 23992773 | 24135610 |
| ENSG00000177409 | 326.5672 | -2.9033876 | 0.6647089 | -4.367908 | 0.0000125 | 0.0100052 | *SAMD9L* | chr7 | 93130056 | 93148385 |
| ENSG00000111801 | 3370.2512 | -0.9813900 | 0.2270701 | -4.321970 | 0.0000155 | 0.0114752 | *BTN3A3* | chr6 | 26440472 | 26453415 |
| ENSG00000188483 | 1524.6048 | 0.7757904 | 0.1805499 | 4.296820 | 0.0000173 | 0.0122727 | *IER5L* | chr9 | 129175552 | 129178261 |
| ENSG00000091483 | 12291.9388 | 0.7333333 | 0.1712344 | 4.282629 | 0.0000185 | 0.0125137 | *FH* | chr1 | 241497603 | 241519755 |
| ENSG00000067066 | 519.5539 | -2.9635602 | 0.6950657 | -4.263713 | 0.0000201 | 0.0130545 | *SP100* | chr2 | 230415942 | 230545606 |
| ENSG00000119285 | 34228.6129 | 0.5079880 | 0.1204164 | 4.218595 | 0.0000246 | 0.0136701 | *HEATR1* | chr1 | 236549005 | 236604516 |
| ENSG00000156535 | 5274.7258 | -0.8792383 | 0.2090495 | -4.205886 | 0.0000260 | 0.0136701 | *CD109* | chr6 | 73695785 | 73828316 |
| ENSG00000180667 | 20315.7445 | 0.6285976 | 0.1482477 | 4.240183 | 0.0000223 | 0.0136701 | *YOD1* | chr1 | 207043849 | 207052980 |
| ENSG00000196663 | 7064.7516 | -0.5217342 | 0.1238737 | -4.211826 | 0.0000253 | 0.0136701 | *TECPR2* | chr14 | 102362941 | 102502477 |
| ENSG00000258659 | 470.5946 | -2.3168870 | 0.5512204 | -4.203195 | 0.0000263 | 0.0136701 | *TRIM34* | chr11 | 5619764 | 5644398 |
| ENSG00000152778 | 6252.0937 | -0.6252050 | 0.1493659 | -4.185727 | 0.0000284 | 0.0142888 | *IFIT5* | chr10 | 89414568 | 89420997 |
| ENSG00000136628 | 44711.8565 | 0.7363871 | 0.1765909 | 4.170017 | 0.0000305 | 0.0148320 | *EPRS1* | chr1 | 219968600 | 220046530 |
| ENSG00000106785 | 10343.1704 | -0.6451561 | 0.1558462 | -4.139698 | 0.0000348 | 0.0156921 | *TRIM14* | chr9 | 98069275 | 98119222 |
| ENSG00000119917 | 904.5428 | -2.4496757 | 0.5921918 | -4.136625 | 0.0000352 | 0.0156921 | *IFIT3* | chr10 | 89327997 | 89340971 |
| ENSG00000168243 | 3526.6625 | 1.0881876 | 0.2623471 | 4.147893 | 0.0000336 | 0.0156921 | *GNG4* | chr1 | 235547685 | 235650754 |
| ENSG00000240184 | 8191.7966 | -1.0559380 | 0.2560556 | -4.123862 | 0.0000373 | 0.0161257 | *PCDHGC3* | chr5 | 141475947 | 141512977 |
| ENSG00000179873 | 107.7020 | -2.5119765 | 0.6164966 | -4.074599 | 0.0000461 | 0.0189021 | *NLRP11* | chr19 | 55785397 | 55836800 |
| ENSG00000112715 | 13995.5603 | 0.9542522 | 0.2350672 | 4.059486 | 0.0000492 | 0.0191596 | *VEGFA* | chr6 | 43770184 | 43786487 |
| ENSG00000116761 | 3510.8257 | 1.3244131 | 0.3259625 | 4.063085 | 0.0000484 | 0.0191596 | *CTH* | chr1 | 70411218 | 70439851 |
| ENSG00000153207 | 29331.2330 | 0.7928481 | 0.1974669 | 4.015094 | 0.0000594 | 0.0225848 | *AHCTF1* | chr1 | 246839098 | 246931948 |
| ENSG00000135916 | 25928.4885 | -0.6507759 | 0.1623845 | -4.007623 | 0.0000613 | 0.0226281 | *ITM2C* | chr2 | 230864639 | 230879248 |
| ENSG00000174282 | 14808.0480 | -0.6622972 | 0.1654340 | -4.003393 | 0.0000624 | 0.0226281 | *ZBTB4* | chr17 | 7459366 | 7484263 |
| ENSG00000165507 | 2823.1858 | -0.8834080 | 0.2221610 | -3.976431 | 0.0000700 | 0.0247760 | *DEPP1* | chr10 | 44970981 | 44978809 |
| ENSG00000229474 | 120.1321 | -1.7028789 | 0.4299254 | -3.960871 | 0.0000747 | 0.0258598 | *PATL2* | chr15 | 44665732 | 44711316 |
| ENSG00000019144 | 13387.2675 | -0.5769927 | 0.1463406 | -3.942807 | 0.0000805 | 0.0272816 | *PHLDB1* | chr11 | 118606440 | 118658031 |
| ENSG00000068079 | 2516.0759 | -1.7839718 | 0.4536301 | -3.932658 | 0.0000840 | 0.0278544 | *IFI35* | chr17 | 43006740 | 43014456 |
| ENSG00000101695 | 6754.5197 | 1.0117457 | 0.2593445 | 3.901166 | 0.0000957 | 0.0295405 | *RNF125* | chr18 | 32018825 | 32073219 |
| ENSG00000108187 | 4275.6116 | -0.5427935 | 0.1395223 | -3.890372 | 0.0001001 | 0.0295405 | *PBLD* | chr10 | 68282660 | 68333049 |
| ENSG00000137955 | 40012.6338 | 0.8051323 | 0.2065704 | 3.897617 | 0.0000971 | 0.0295405 | *RABGGTB* | chr1 | 75786197 | 75795086 |
| ENSG00000143847 | 3471.3456 | 0.6376910 | 0.1633804 | 3.903106 | 0.0000950 | 0.0295405 | *PPFIA4* | chr1 | 203026498 | 203078740 |
| ENSG00000146352 | 1808.2053 | 1.2547290 | 0.3225980 | 3.889450 | 0.0001005 | 0.0295405 | *CLVS2* | chr6 | 122996235 | 123072925 |
| ENSG00000166710 | 48412.8956 | -0.5281384 | 0.1352999 | -3.903466 | 0.0000948 | 0.0295405 | *B2M* | chr15 | 44711487 | 44718877 |
| ENSG00000196544 | 2007.1023 | -0.5512961 | 0.1419176 | -3.884622 | 0.0001025 | 0.0295757 | *BORCS6* | chr17 | 8188345 | 8190180 |
| ENSG00000150627 | 4054.6870 | 0.5492517 | 0.1416368 | 3.877888 | 0.0001054 | 0.0296446 | *WDR17* | chr4 | 176065834 | 176182818 |
| ENSG00000196126 | 949.2674 | -1.0975761 | 0.2835455 | -3.870899 | 0.0001084 | 0.0296446 | *HLA-DRB1* | chr6 | 32578769 | 32589848 |
| ENSG00000250722 | 8951.2092 | -1.4111017 | 0.3644652 | -3.871704 | 0.0001081 | 0.0296446 | *SELENOP* | chr5 | 42799880 | 42887392 |
| ENSG00000198860 | 14947.9963 | 0.6411183 | 0.1660288 | 3.861488 | 0.0001127 | 0.0302789 | *TSEN15* | chr1 | 184051651 | 184123978 |
| ENSG00000205413 | 6546.4473 | -1.4921379 | 0.3890323 | -3.835512 | 0.0001253 | 0.0325433 | *SAMD9* | chr7 | 93099513 | 93118023 |
| ENSG00000178685 | 942.8927 | -1.8479533 | 0.4835863 | -3.821352 | 0.0001327 | 0.0337695 | *PARP10* | chr8 | 143977153 | 144012772 |
| ENSG00000243811 | 1445.5065 | -1.0245035 | 0.2685898 | -3.814380 | 0.0001365 | 0.0337695 | *APOBEC3D* | chr22 | 39021113 | 39033277 |
| ENSG00000106868 | 4965.6130 | -0.7764799 | 0.2039395 | -3.807403 | 0.0001404 | 0.0341935 | *SUSD1* | chr9 | 112040783 | 112175297 |
| ENSG00000120820 | 2498.4605 | -0.6672998 | 0.1756312 | -3.799438 | 0.0001450 | 0.0342412 | *GLT8D2* | chr12 | 103988984 | 104064183 |
| ENSG00000186063 | 19631.5674 | 0.5765695 | 0.1516559 | 3.801828 | 0.0001436 | 0.0342412 | *AIDA* | chr1 | 222668013 | 222713210 |
| ENSG00000185885 | 2706.1898 | -1.7784731 | 0.4713799 | -3.772908 | 0.0001614 | 0.0375284 | *IFITM1* | chr11 | 313506 | 315272 |
| ENSG00000102313 | 606.4737 | -1.6075749 | 0.4284709 | -3.751888 | 0.0001755 | 0.0390705 | *ITIH6* | chrX | 54748918 | 54798255 |
| ENSG00000159403 | 3083.7176 | -1.0342706 | 0.2772191 | -3.730878 | 0.0001908 | 0.0412980 | *C1R* | chr12 | 7080214 | 7092540 |
| ENSG00000163938 | 61109.6997 | 0.6140843 | 0.1654485 | 3.711634 | 0.0002059 | 0.0426063 | *GNL3* | chr3 | 52681156 | 52694497 |
| ENSG00000187134 | 208.9296 | -2.7196922 | 0.7326655 | -3.712051 | 0.0002056 | 0.0426063 | *AKR1C1* | chr10 | 4963253 | 4983283 |
| ENSG00000146592 | 19942.2661 | 0.7251710 | 0.1964353 | 3.691653 | 0.0002228 | 0.0442008 | *CREB5* | chr7 | 28299321 | 28825894 |
| ENSG00000156042 | 2222.3303 | -0.6337066 | 0.1715045 | -3.694984 | 0.0002199 | 0.0442008 | *CFAP70* | chr10 | 73253759 | 73358859 |
| ENSG00000162714 | 7173.9395 | 0.7951591 | 0.2157360 | 3.685796 | 0.0002280 | 0.0442008 | *ZNF496* | chr1 | 247297412 | 247331846 |
| ENSG00000189362 | 2726.1388 | -1.0962219 | 0.2970811 | -3.689975 | 0.0002243 | 0.0442008 | *NEMP2* | chr2 | 190504338 | 190534722 |
| ENSG00000156587 | 5294.5362 | -0.9646697 | 0.2633871 | -3.662555 | 0.0002497 | 0.0467155 | *UBE2L6* | chr11 | 57551656 | 57568284 |
| ENSG00000223839 | 855.5723 | -1.5117875 | 0.4130113 | -3.660402 | 0.0002518 | 0.0467155 | *FAM95B1* | chr9 | 40321299 | 40329221 |
| ENSG00000154065 | 1738.9205 | 0.5635157 | 0.1545298 | 3.646648 | 0.0002657 | 0.0487075 | *ANKRD29* | chr18 | 23598926 | 23662911 |
