# Supplementary material for: Low-grade glioma risk SNP rs11706832 is associated with type I interferon response pathway genes in cell lines
Source: Sci Rep. 2023 Apr 25;13:6777. doi: 10.1038/s41598-023-33923-4 (PMC10130147; doi:10.1038/s41598-023-33923-4)
Supplement: Supplementary file 9 — Supplementary Table S3. [file 41598_2023_33923_MOESM9_ESM.docx]

# S3. Differential expression of mitochondrial genes *in cell lines, C vs. A at SNP position*

**baseMean**

mean normalized count across all samples

**log2FoldChange**

log_2_ fold change

**lfcSE**

standard error of log_2_ fold change

**stat**

Wald statistic

**pvalue**

p-value from Wald test

**padj**

Benjamini-Hochberg corrected p-value

Sorted on **Pvalue**

|  | baseMean | log2FoldChange | lfcSE | stat | pvalue | padj | gene_name |
| --- | --- | --- | --- | --- | --- | --- | --- |
| ENSG00000198763 | 2678656.0 | 0.5604534 | 0.1751910 | 3.199100 | 0.0013786 | 0.0049486 | *MT-ND2* |
| ENSG00000228253 | 911217.5 | 0.4500159 | 0.1444218 | 3.115984 | 0.0018333 | 0.0049486 | *MT-ATP8* |
| ENSG00000198899 | 3554376.3 | 0.4886158 | 0.1398501 | 3.493853 | 0.0004761 | 0.0049486 | *MT-ATP6* |
| ENSG00000198938 | 2684031.7 | 0.3720720 | 0.1199895 | 3.100873 | 0.0019295 | 0.0049486 | *MT-CO3* |
| ENSG00000198840 | 421739.8 | 0.5756639 | 0.1832545 | 3.141336 | 0.0016818 | 0.0049486 | *MT-ND3* |
| ENSG00000198886 | 5080787.4 | 0.4857886 | 0.1592445 | 3.050583 | 0.0022840 | 0.0049486 | *MT-ND4* |
| ENSG00000212907 | 664800.3 | 0.4761377 | 0.1601009 | 2.973985 | 0.0029396 | 0.0054593 | *MT-ND4L* |
| ENSG00000198712 | 4120064.7 | 0.3711787 | 0.1275022 | 2.911154 | 0.0036010 | 0.0058516 | *MT-CO2* |
| ENSG00000198786 | 4266163.4 | 0.4922449 | 0.2054479 | 2.395960 | 0.0165769 | 0.0239444 | *MT-ND5* |
| ENSG00000198727 | 2682130.0 | 0.2564183 | 0.1435814 | 1.785874 | 0.0741196 | 0.0963555 | *MT-CYB* |
| ENSG00000198804 | 6267498.5 | 0.3090598 | 0.1841289 | 1.678497 | 0.0932500 | 0.1102046 | *MT-CO1* |
| ENSG00000198888 | 2492777.1 | 0.2377798 | 0.1889120 | 1.258681 | 0.2081458 | 0.2254912 | *MT-ND1* |
| ENSG00000198695 | 131191.8 | -0.2054941 | 0.2015841 | -1.019397 | 0.3080147 | 0.3080147 | *MT-ND6* |
