# Supplementary material for: Low-grade glioma risk SNP rs11706832 is associated with type I interferon response pathway genes in cell lines
Source: Sci Rep. 2023 Apr 25;13:6777. doi: 10.1038/s41598-023-33923-4 (PMC10130147; doi:10.1038/s41598-023-33923-4)
Supplement: Supplementary file 10 — Supplementary Table S4. [file 41598_2023_33923_MOESM10_ESM.docx]

# S4. Enriched terms in GO Biological pathways in cell lines

**GeneRatio**

Ratio of number of genes from tested gene set that are involved in pathway and number of genes from gene set that are involved in any pathway

**BgRatio**

Ratio of number of genes in pathway and number of genes in any pathway

**Pvalue**

p-value from hypergeometric test

**p.adjust**

Benjamini-Hochberg corrected p-value

**geneID**

Genes from tested gene set found in pathway

**Count**

Number of genes from tested gene set in pathway

|  | GeneRatio | BgRatio | pvalue | p.adjust | geneID | Count |
| --- | --- | --- | --- | --- | --- | --- |
| cellular response to type I interferon (GO:0071357) | 7/61 | 65/14937 | 0.0000000 | 0.0000025 | *OAS1/IFIT2/SP100/IFIT5/IFIT3/IFI35/IFITM1* | 7 |
| type I interferon signaling pathway (GO:0060337) | 7/61 | 65/14937 | 0.0000000 | 0.0000025 | *OAS1/IFIT2/SP100/IFIT5/IFIT3/IFI35/IFITM1* | 7 |
| interferon-gamma-mediated signaling pathway (GO:0060333) | 6/61 | 68/14937 | 0.0000003 | 0.0000705 | *OAS1/HLA-DRB5/SP100/TRIM34/B2M/HLA-DRB1* | 6 |
| cellular response to interferon-gamma (GO:0071346) | 7/61 | 121/14937 | 0.0000006 | 0.0000899 | *OAS1/HLA-DRB5/SP100/TRIM34/EPRS1/B2M/HLA-DRB1* | 7 |
| defense response to symbiont (GO:0140546) | 7/61 | 124/14937 | 0.0000007 | 0.0000899 | *TLR3/OAS1/IFIT2/IFIT5/IFIT3/APOBEC3D/IFITM1* | 7 |
| defense response to virus (GO:0051607) | 7/61 | 133/14937 | 0.0000011 | 0.0001203 | *TLR3/OAS1/IFIT2/IFIT5/IFIT3/APOBEC3D/IFITM1* | 7 |
| negative regulation of viral genome replication (GO:0045071) | 5/61 | 54/14937 | 0.0000026 | 0.0002428 | *OAS1/IFIT5/PARP10/APOBEC3D/IFITM1* | 5 |
| innate immune response (GO:0045087) | 9/61 | 302/14937 | 0.0000035 | 0.0002816 | *TRIM17/TLR3/OAS1/CXCL16/SP100/TRIM34/TRIM14/B2M/IFITM1* | 9 |
| negative regulation of viral process (GO:0048525) | 5/61 | 70/14937 | 0.0000095 | 0.0006875 | *OAS1/IFIT5/TRIM14/PARP10/IFITM1* | 5 |
| regulation of viral entry into host cell (GO:0046596) | 4/61 | 39/14937 | 0.0000186 | 0.0012129 | *TRIM34/TRIM14/HLA-DRB1/IFITM1* | 4 |
| regulation of viral genome replication (GO:0045069) | 4/61 | 67/14937 | 0.0001592 | 0.0094353 | *OAS1/IFIT5/PARP10/IFITM1* | 4 |
| response to interferon-gamma (GO:0034341) | 4/61 | 80/14937 | 0.0003157 | 0.0171545 | *CXCL16/SP100/EPRS1/IFITM1* | 4 |
| negative regulation of cell population proliferation (GO:0008285) | 7/61 | 379/14937 | 0.0008596 | 0.0431119 | *NMI/PTPN6/IFIT3/IFI35/B2M/PARP10/IFITM1* | 7 |
| toll-like receptor 3 signaling pathway (GO:0034138) | 2/61 | 12/14937 | 0.0010546 | 0.0491144 | *TLR3/OAS1* | 2 |
