# Supplementary material for: Low-grade glioma risk SNP rs11706832 is associated with type I interferon response pathway genes in cell lines
Source: Sci Rep. 2023 Apr 25;13:6777. doi: 10.1038/s41598-023-33923-4 (PMC10130147; doi:10.1038/s41598-023-33923-4)
Supplement: Supplementary file 11 — Supplementary Table S5. [file 41598_2023_33923_MOESM11_ESM.docx]

# S5. Differential expressed genes *TCGA LGG tumor samples* conditioned on genotype on SNP position

**baseMean**

mean normalized count across all samples

**log2FoldChange**

log_2_ fold change

**lfcSE**

standard error of log_2_ fold change

**pvalue**

p-value from Likelihood-ratio test

**padj**

Benjamini-Hochberg corrected p-value

**pvalue1**

p-value using Wald test on CC vs AA genotype

**pvalue2**

p-value using Wald test on CA vs AA genotype

**pvalue3**

p-value using Wald test on CC vs CA genotype

Sorted on **pvalue**

|  | baseMean | log2FoldChange | lfcSE | stat | pvalue | padj | pvalue1 | padj1 | pvalue2 | padj2 | pvalue3 | padj3 | log2FoldChange1 | log2FoldChange2 | log2FoldChange3 | gene_name | chrom | start | end |
| --- | --- | --- | --- | --- | --- | --- | --- | --- | --- | --- | --- | --- | --- | --- | --- | --- | --- | --- | --- |
| ENSG00000198744 | 342,16521 | -1,605665 | 0,2535225 | 76,11086 | 0,00E+00 | 0 | 0 | 0,0000012 | 0 | 0 | 0,2580404 | 0,6493583 | -1,605665 | -1,8395094 | 0,2338443 | *MTCO3P12* | chr1 | 634376 | 634922 |
| ENSG00000253537 | 117,28896 | 0,256234 | 0,2153823 | 53,26911 | 0,00E+00 | 0 | 0,234176 | 0,9995928 | 0 | 0,000002 | 0 | 0,0000779 | 0,256234 | 1,2677715 | -1,0115375 | *PCDHGA7* | chr5 | 141382739 | 141512975 |
| ENSG00000253159 | 754,18636 | 2,6919885 | 0,3330853 | 51,99323 | 0,00E+00 | 0 | 0 | 0 | 0 | 0,0000001 | 0,0337329 | 0,3400539 | 2,6919885 | 2,1160434 | 0,5759451 | *PCDHGA12* | chr5 | 141430507 | 141512975 |
| ENSG00000073792 | 57,05409 | -1,2773142 | 0,1996993 | 51,23392 | 0,00E+00 | 0 | 0 | 0,0000011 | 0 | 0,0000557 | 0,1953967 | 0,596075 | -1,2773142 | -1,0656554 | -0,2116588 | *IGF2BP2* | chr3 | 185643130 | 185825042 |
| ENSG00000012223 | 124,90124 | 1,3299982 | 0,2910759 | 38,4179 | 0,00E+00 | 0,000016 | 0,0000049 | 0,0099564 | 0,965902 | 0,9979279 | 0 | 0,0001392 | 1,3299982 | 0,0116385 | 1,3183597 | *LTF* | chr3 | 46435645 | 46485234 |
| ENSG00000261934 | 810,33562 | 2,1520579 | 0,3189163 | 38,38348 | 0,00E+00 | 0,000016 | 0 | 0,0000002 | 0 | 0,0000947 | 0,0612955 | 0,4141737 | 2,1520579 | 1,6659257 | 0,4861322 | *PCDHGA9* | chr5 | 141402932 | 141512979 |
| ENSG00000167244 | 1346,74014 | 0,6135942 | 0,1645038 | 35,37384 | 0,00E+00 | 0,0000617 | 0,0001915 | 0,1343224 | 0,3231841 | 0,9516154 | 0 | 0,0000779 | 0,6135942 | -0,1519028 | 0,7654969 | *IGF2* | chr11 | 2129112 | 2141238 |
| ENSG00000251664 | 35,45898 | -0,6524912 | 0,2285576 | 32,25181 | 1,00E-07 | 0,0002573 | 0,0043061 | 0,5554937 | 0,0408426 | 0,8701974 | 0 | 0,0000779 | -0,6524912 | 0,4348723 | -1,0873635 | *PCDHA12* | chr5 | 140875302 | 141012347 |
| ENSG00000262902 | 116,05407 | 0,5699523 | 0,1760591 | 29,13365 | 5,00E-07 | 0,0010873 | 0,0012068 | 0,3427797 | 0,2739528 | 0,9346459 | 0,0000002 | 0,000703 | 0,5699523 | -0,1802411 | 0,7501934 | *MTCO1P40* | chr17 | 53105734 | 53106358 |
| ENSG00000042832 | 127,54232 | -0,5186165 | 0,2276885 | 28,05384 | 8,00E-07 | 0,0016401 | 0,0227417 | 0,8258501 | 0,0000004 | 0,0015491 | 0,002774 | 0,1816307 | -0,5186165 | -1,0744467 | 0,5558302 | *TG* | chr8 | 132866958 | 133134903 |
| ENSG00000187608 | 2612,1569 | -0,2101482 | 0,2680343 | 27,91019 | 9,00E-07 | 0,0016401 | 0,4330198 | 0,9995928 | 0,0000056 | 0,0115149 | 0,0000216 | 0,0407504 | -0,2101482 | -1,1379688 | 0,9278206 | *ISG15* | chr1 | 1001138 | 1014540 |
| ENSG00000111331 | 1880,7498 | -0,8213639 | 0,1854785 | 27,4134 | 1,10E-06 | 0,001819 | 0,0000095 | 0,0175591 | 0,0000043 | 0,0098313 | 0,8719466 | 0,9624562 | -0,8213639 | -0,796998 | -0,0243659 | *OAS3* | chr12 | 112938444 | 112973251 |
| ENSG00000113263 | 31,78443 | -0,0274201 | 0,2796804 | 27,36903 | 1,10E-06 | 0,001819 | 0,9218998 | 0,9995928 | 0,0000847 | 0,0836616 | 0,0000036 | 0,0125112 | -0,0274201 | 1,0247935 | -1,0522136 | *ITK* | chr5 | 157142933 | 157255185 |
| ENSG00000168528 | 237,99514 | -1,0362007 | 0,2058527 | 26,08181 | 2,20E-06 | 0,0032149 | 0,0000005 | 0,0015755 | 0,0048496 | 0,6481749 | 0,0032168 | 0,1869412 | -1,0362007 | -0,5416395 | -0,4945612 | *SERINC2* | chr1 | 31409565 | 31434680 |
| ENSG00000128564 | 2898,42053 | -1,5521261 | 0,3113599 | 25,64848 | 2,70E-06 | 0,0034936 | 0,0000006 | 0,0015755 | 0,0058354 | 0,661285 | 0,003121 | 0,1869412 | -1,5521261 | -0,8021808 | -0,7499453 | *VGF* | chr7 | 101162509 | 101165569 |
| ENSG00000152760 | 48,87617 | 1,3162913 | 0,2631795 | 25,73117 | 2,60E-06 | 0,0034936 | 0,0000006 | 0,0015755 | 0,0131853 | 0,7705867 | 0,0009601 | 0,1455223 | 1,3162913 | 0,6106925 | 0,7055988 | *TCTEX1D1* | chr1 | 66752459 | 66779047 |
| ENSG00000073282 | 17,07091 | -0,5950771 | 0,1752931 | 23,84125 | 6,70E-06 | 0,0081168 | 0,0006869 | 0,2972814 | 0,0000027 | 0,0079749 | 0,2289584 | 0,6246059 | -0,5950771 | -0,7690619 | 0,1739848 | *TP63* | chr3 | 189631389 | 189897276 |
| ENSG00000178429 | 74,79509 | -0,5073947 | 0,2504533 | 23,41529 | 8,20E-06 | 0,0094854 | 0,0427745 | 0,9272056 | 0,0000042 | 0,0098313 | 0,0052742 | 0,2183137 | -0,5073947 | -1,0778349 | 0,5704402 | *RPS3AP5* | chr10 | 84560443 | 84561263 |
| ENSG00000176601 | 59,68658 | 1,5431954 | 0,3312017 | 22,34456 | 1,41E-05 | 0,0153489 | 0,0000032 | 0,0071681 | 0,0225283 | 0,8099274 | 0,001898 | 0,164734 | 1,5431954 | 0,7068757 | 0,8363197 | *MAP3K19* | chr2 | 134964485 | 135047468 |
| ENSG00000205293 | 649,87696 | 2,4704033 | 0,4912337 | 21,99671 | 1,67E-05 | 0,0173515 | 0,0000005 | 0,0015755 | 0,0011711 | 0,449895 | 0,0143182 | 0,2723567 | 2,4704033 | 1,4902002 | 0,980203 | *LINC01602* | chr8 | 57855500 | 57984126 |
| ENSG00000089127 | 813,99163 | -0,4252016 | 0,2018688 | 21,57127 | 2,07E-05 | 0,0204423 | 0,035176 | 0,913948 | 0,0000084 | 0,0157988 | 0,0115884 | 0,2589057 | -0,4252016 | -0,8405692 | 0,4153676 | *OAS1* | chr12 | 112906783 | 112933222 |
| ENSG00000148848 | 142,72641 | -0,0185751 | 0,1913512 | 21,2382 | 2,44E-05 | 0,0230491 | 0,9226682 | 0,9995928 | 0,0005669 | 0,3448049 | 0,0000464 | 0,0739686 | -0,0185751 | 0,6159866 | -0,6345617 | *ADAM12* | chr10 | 126012381 | 126388455 |
| ENSG00000105929 | 29,46488 | 0,8322078 | 0,2733275 | 20,89246 | 2,91E-05 | 0,0231835 | 0,0023289 | 0,4246145 | 0,6250307 | 0,9761753 | 0,0000169 | 0,0350249 | 0,8322078 | -0,1251925 | 0,9574003 | *ATP6V0A4* | chr7 | 138706294 | 138799560 |
| ENSG00000134321 | 556,81153 | -0,6262312 | 0,1889356 | 20,97353 | 2,79E-05 | 0,0231835 | 0,000918 | 0,3271165 | 0,0000138 | 0,0238848 | 0,3590155 | 0,7244647 | -0,6262312 | -0,7675026 | 0,1412714 | *RSAD2* | chr2 | 6865806 | 6898239 |
| ENSG00000204963 | 67,36376 | 0,7106334 | 0,250872 | 20,9404 | 2,84E-05 | 0,0231835 | 0,0046163 | 0,5554937 | 0,4268946 | 0,9593591 | 0,0000114 | 0,0265165 | 0,7106334 | -0,186528 | 0,8971614 | *PCDHA7* | chr5 | 140834248 | 141012347 |
| ENSG00000229604 | 33,11314 | -0,2513703 | 0,2717685 | 20,90146 | 2,89E-05 | 0,0231835 | 0,3549958 | 0,9995928 | 0,0045337 | 0,6476385 | 0,0000114 | 0,0265165 | -0,2513703 | 0,7190268 | -0,9703971 | *MTATP8P2* | chr2 | 87824942 | 87825143 |
| ENSG00000110887 | 209,11368 | 1,369276 | 0,3776318 | 20,69253 | 3,21E-05 | 0,0246719 | 0,0002879 | 0,1793424 | 0,6434555 | 0,9792227 | 0,0000887 | 0,0876516 | 1,369276 | 0,1634012 | 1,2058748 | *DAO* | chr12 | 108858932 | 108901043 |
| ENSG00000163885 | 17,57782 | 0,7194606 | 0,1874206 | 19,92669 | 4,71E-05 | 0,034015 | 0,0001237 | 0,1023045 | 0,3795166 | 0,9540093 | 0,0001968 | 0,0933283 | 0,7194606 | 0,1549066 | 0,564554 | *CFAP100* | chr3 | 126394909 | 126436556 |
| ENSG00000165507 | 2169,68704 | 0,4439042 | 0,1925406 | 19,84706 | 4,90E-05 | 0,034015 | 0,0211381 | 0,8161798 | 0,1744148 | 0,9241156 | 0,0000115 | 0,0265165 | 0,4439042 | -0,2443978 | 0,688302 | *DEPP1* | chr10 | 44970981 | 44978809 |
| ENSG00000259040 | 23,15727 | 0,7091451 | 0,1832562 | 19,83954 | 4,92E-05 | 0,034015 | 0,000109 | 0,098658 | 0,343991 | 0,9540009 | 0,000234 | 0,0990762 | 0,7091451 | 0,1628691 | 0,546276 | *BLOC1S5-TXNDC5* | chr6 | 7881522 | 8064364 |
| ENSG00000131055 | 57,43283 | 0,4896853 | 0,1537309 | 19,69455 | 5,29E-05 | 0,0353927 | 0,0014458 | 0,3501147 | 0,8350542 | 0,985266 | 0,0000327 | 0,0564502 | 0,4896853 | -0,0299904 | 0,5196758 | *COX4I2* | chr20 | 31637912 | 31645006 |
| ENSG00000157601 | 1911,91714 | -0,5842342 | 0,1633353 | 19,53746 | 5,72E-05 | 0,0370882 | 0,0003477 | 0,1911454 | 0,0000472 | 0,0576052 | 0,7817693 | 0,930659 | -0,5842342 | -0,6211099 | 0,0368757 | *MX1* | chr21 | 41420304 | 41459214 |
| ENSG00000241644 | 91,45785 | 0,9614501 | 0,2716045 | 19,16271 | 6,90E-05 | 0,043376 | 0,0004003 | 0,2142735 | 0,6246383 | 0,9761753 | 0,0001536 | 0,0916584 | 0,9614501 | 0,1243006 | 0,8371495 | *INMT* | chr7 | 30697985 | 30757602 |
| ENSG00000134326 | 407,32306 | -0,6575207 | 0,1743584 | 19,05966 | 7,27E-05 | 0,0443262 | 0,0001625 | 0,1224515 | 0,0001245 | 0,1173795 | 0,8200553 | 0,9456748 | -0,6575207 | -0,6251844 | -0,0323363 | *CMPK2* | chr2 | 6840570 | 6866635 |
| ENSG00000141255 | 39,43614 | -0,5329938 | 0,1697397 | 18,87474 | 7,97E-05 | 0,0459191 | 0,0016891 | 0,3817546 | 0,0000315 | 0,0466255 | 0,3598669 | 0,7248427 | -0,5329938 | -0,6602791 | 0,1272853 | *SPATA22* | chr17 | 3440019 | 3513852 |
| ENSG00000241278 | 54,19691 | -0,785509 | 0,1855777 | 18,92504 | 7,77E-05 | 0,0459191 | 0,0000231 | 0,0391266 | 0,0022051 | 0,5427946 | 0,0921997 | 0,4752371 | -0,785509 | -0,530079 | -0,25543 | *ENPP7P4* | chr3 | 125848223 | 125909372 |
| ENSG00000164611 | 385,62044 | -0,016861 | 0,202798 | 18,52572 | 9,49E-05 | 0,0499719 | 0,9337387 | 0,9995928 | 0,0007748 | 0,368176 | 0,0001748 | 0,0916584 | -0,016861 | -0,6372551 | 0,6203941 | *PTTG1* | chr5 | 160421855 | 160428739 |
| ENSG00000185745 | 4000,2495 | -0,3091364 | 0,1779097 | 18,62271 | 9,04E-05 | 0,0499719 | 0,0822808 | 0,9839355 | 0,000041 | 0,0563763 | 0,0101194 | 0,2549309 | -0,3091364 | -0,6820028 | 0,3728664 | *IFIT1* | chr10 | 89392546 | 89406487 |
| ENSG00000204118 | 44,81322 | 0,5077611 | 0,3029766 | 18,49486 | 9,64E-05 | 0,0499719 | 0,0937561 | 0,9895374 | 0,0000287 | 0,0458364 | 0,0060127 | 0,2239254 | 0,5077611 | 1,1837919 | -0,6760308 | *NAP1L6P* | chrX | 73126037 | 73128080 |
| ENSG00000204961 | 86,97741 | -0,5804788 | 0,3263251 | 18,57867 | 9,24E-05 | 0,0499719 | 0,0752667 | 0,9717407 | 0,0000435 | 0,0563763 | 0,0122617 | 0,263299 | -0,5804788 | -1,2472244 | 0,6667456 | *PCDHA9* | chr5 | 140847772 | 141012347 |
