# Supplementary material for: Low-grade glioma risk SNP rs11706832 is associated with type I interferon response pathway genes in cell lines
Source: Sci Rep. 2023 Apr 25;13:6777. doi: 10.1038/s41598-023-33923-4 (PMC10130147; doi:10.1038/s41598-023-33923-4)
Supplement: Supplementary file 12 — Supplementary Table S6. [file 41598_2023_33923_MOESM12_ESM.docx]

# S6. Differential expression of mitochondrial genes *in TCGA LGG tumor samples*

**baseMean**

mean normalized count across all samples

**log2FoldChange**

log_2_ fold change

**lfcSE**

standard error of log_2_ fold change

**pvalue**

p-value from Likelihood-ratio test

**padj**

Benjamini-Hochberg corrected p-value

**pvalue1**

p-value using Wald test on CC vs AA genotype

**pvalue2**

p-value using Wald test on CA vs AA genotype

**pvalue3**

p-value using Wald test on CC vs CA genotype

Sorted on **pvalue**

|  | baseMean | log2FoldChange | lfcSE | stat | pvalue | padj | pvalue1 | padj1 | pvalue2 | padj2 | pvalue3 | padj3 | log2FoldChange1 | log2FoldChange2 | log2FoldChange3 | gene_name |
| --- | --- | --- | --- | --- | --- | --- | --- | --- | --- | --- | --- | --- | --- | --- | --- | --- |
| ENSG00000198763 | 262056,85 | -0,0835414 | 0,1183485 | 1,7171428 | 0,4237671 | 0,9853271 | 0,4802544 | 0,9995928 | 0,6971178 | 0,9805357 | 0,1893263 | 0,5897195 | -0,0835414 | 0,0430481 | -0,1265895 | *MT-ND2* |
| ENSG00000228253 | 34210,27 | 0,0439442 | 0,1429747 | 0,5356935 | 0,765025 | 0,9853271 | 0,758572 | 0,9995928 | 0,4799098 | 0,9681264 | 0,6650168 | 0,8852665 | 0,0439442 | 0,0943935 | -0,0504492 | *MT-ATP8* |
| ENSG00000198899 | 284518,23 | 0,0218214 | 0,111358 | 0,0560489 | 0,9723646 | 0,9853271 | 0,8446438 | 0,9995928 | 0,9794664 | 0,9979279 | 0,8329284 | 0,9505566 | 0,0218214 | 0,0026785 | 0,0191428 | *MT-ATP6* |
| ENSG00000198938 | 403097,17 | -0,070408 | 0,1041438 | 0,7893788 | 0,6738893 | 0,9853271 | 0,498999 | 0,9995928 | 0,9958106 | 0,9996637 | 0,4033582 | 0,7509898 | -0,070408 | 0,000511 | -0,0709191 | *MT-CO3* |
| ENSG00000198840 | 104456,67 | 0,0478179 | 0,1135372 | 0,2631388 | 0,8767184 | 0,9853271 | 0,6736345 | 0,9995928 | 0,6184299 | 0,9758925 | 0,9566344 | 0,986941 | 0,0478179 | 0,0528491 | -0,0050312 | *MT-ND3* |
| ENSG00000198886 | 791409,65 | -0,0444629 | 0,1140807 | 0,1525886 | 0,9265435 | 0,9853271 | 0,6967218 | 0,9995928 | 0,7909115 | 0,9843401 | 0,8616919 | 0,9596655 | -0,0444629 | -0,0282663 | -0,0161966 | *MT-ND4* |
| ENSG00000212907 | 44435,86 | 0,001555 | 0,1319399 | 0,6782269 | 0,7124016 | 0,9853271 | 0,9905966 | 0,9995928 | 0,5252854 | 0,969791 | 0,4752183 | 0,794715 | 0,001555 | 0,0783254 | -0,0767704 | *MT-ND4L* |
| ENSG00000198712 | 381904,82 | -0,0432273 | 0,1121144 | 0,1498556 | 0,9278105 | 0,9853271 | 0,6998193 | 0,9995928 | 0,7866115 | 0,9839927 | 0,8707713 | 0,9621917 | -0,0432273 | -0,0283644 | -0,0148629 | *MT-CO2* |
| ENSG00000198786 | 290099,96 | -0,0207352 | 0,1208716 | 0,0295632 | 0,9853271 | 0,9853271 | 0,8637935 | 0,9995928 | 0,905679 | 0,992659 | 0,9405142 | 0,9835015 | -0,0207352 | -0,0133848 | -0,0073504 | *MT-ND5* |
| ENSG00000198727 | 338112,95 | -0,0833665 | 0,1240426 | 0,567108 | 0,7531024 | 0,9853271 | 0,5015326 | 0,9995928 | 0,8627729 | 0,9878221 | 0,5309834 | 0,8251934 | -0,0833665 | -0,0200369 | -0,0633296 | *MT-CYB* |
| ENSG00000198804 | 939359,18 | -0,1026408 | 0,1058995 | 0,9503191 | 0,6217858 | 0,9853271 | 0,3324313 | 0,9995928 | 0,4969173 | 0,9689496 | 0,6816001 | 0,8925614 | -0,1026408 | -0,067234 | -0,0354068 | *MT-CO1* |
| ENSG00000198888 | 247489,11 | -0,0939906 | 0,1199191 | 2,2294239 | 0,3280098 | 0,9853271 | 0,4331673 | 0,9995928 | 0,640444 | 0,9788065 | 0,1342761 | 0,5291458 | -0,0939906 | 0,0523457 | -0,1463363 | *MT-ND1* |
| ENSG00000198695 | 129849,79 | 0,0508634 | 0,1434692 | 1,6451525 | 0,4392984 | 0,9853271 | 0,7229456 | 0,9995928 | 0,4777195 | 0,9677269 | 0,2115722 | 0,6107024 | 0,0508634 | -0,095193 | 0,1460564 | *MT-ND6* |
