# Supplementary material for: Low-grade glioma risk SNP rs11706832 is associated with type I interferon response pathway genes in cell lines
Source: Sci Rep. 2023 Apr 25;13:6777. doi: 10.1038/s41598-023-33923-4 (PMC10130147; doi:10.1038/s41598-023-33923-4)
Supplement: Supplementary file 13 — Supplementary Table S7. [file 41598_2023_33923_MOESM13_ESM.docx]

# S7. Enriched terms in GO Biological pathways in TCGA LGG tumor samples

**GeneRatio**

Ratio of number of genes from tested gene set that are involved in pathway and number of genes from gene set that are involved in any pathway

**BgRatio**

Ratio of number of genes in pathway and number of genes in any pathway

**Pvalue**

p-value from hypergeometric test

**p.adjust**

Benjamini-Hochberg corrected p-value

**geneID**

Genes from tested gene set found in pathway

**Count**

Number of genes from tested gene set in pathway

|  | GeneRatio | BgRatio | pvalue | p.adjust | qvalue | geneID | Count |
| --- | --- | --- | --- | --- | --- | --- | --- |
| negative regulation of viral genome replication (GO:0045071) | 7/24 | 54/14937 | 0.0000000 | 0.0000000 | 0.0000000 | *LTF/ISG15/OAS3/OAS1/RSAD2/MX1/IFIT1* | 7 |
| regulation of viral genome replication (GO:0045069) | 7/24 | 67/14937 | 0.0000000 | 0.0000000 | 0.0000000 | *LTF/ISG15/OAS3/OAS1/RSAD2/MX1/IFIT1* | 7 |
| negative regulation of viral process (GO:0048525) | 7/24 | 70/14937 | 0.0000000 | 0.0000000 | 0.0000000 | *LTF/ISG15/OAS3/OAS1/RSAD2/MX1/IFIT1* | 7 |
| cellular response to type I interferon (GO:0071357) | 6/24 | 65/14937 | 0.0000000 | 0.0000000 | 0.0000000 | *ISG15/OAS3/OAS1/RSAD2/MX1/IFIT1* | 6 |
| type I interferon signaling pathway (GO:0060337) | 6/24 | 65/14937 | 0.0000000 | 0.0000000 | 0.0000000 | *ISG15/OAS3/OAS1/RSAD2/MX1/IFIT1* | 6 |
| defense response to symbiont (GO:0140546) | 6/24 | 124/14937 | 0.0000000 | 0.0000017 | 0.0000011 | *ISG15/OAS3/OAS1/RSAD2/MX1/IFIT1* | 6 |
| defense response to virus (GO:0051607) | 6/24 | 133/14937 | 0.0000001 | 0.0000023 | 0.0000014 | *ISG15/OAS3/OAS1/RSAD2/MX1/IFIT1* | 6 |
| antiviral innate immune response (GO:0140374) | 3/24 | 14/14937 | 0.0000013 | 0.0000492 | 0.0000311 | *OAS1/MX1/IFIT1* | 3 |
| negative regulation of type I interferon-mediated signaling pathway (GO:0060339) | 3/24 | 16/14937 | 0.0000020 | 0.0000671 | 0.0000424 | *ISG15/OAS3/OAS1* | 3 |
| regulation of type I interferon-mediated signaling pathway (GO:0060338) | 3/24 | 30/14937 | 0.0000144 | 0.0004315 | 0.0002725 | *ISG15/OAS3/OAS1* | 3 |
| positive regulation of interferon-beta production (GO:0032728) | 3/24 | 36/14937 | 0.0000251 | 0.0006854 | 0.0004329 | *ISG15/OAS3/OAS1* | 3 |
| negative regulation of innate immune response (GO:0045824) | 3/24 | 38/14937 | 0.0000296 | 0.0007408 | 0.0004679 | *ISG15/OAS3/OAS1* | 3 |
| regulation of interferon-beta production (GO:0032648) | 3/24 | 49/14937 | 0.0000640 | 0.0014762 | 0.0009323 | *ISG15/OAS3/OAS1* | 3 |
| regulation of cytokine production (GO:0001817) | 4/24 | 150/14937 | 0.0000888 | 0.0018103 | 0.0011433 | *IGF2BP2/LTF/ISG15/ITK* | 4 |
| negative regulation of cytokine-mediated signaling pathway (GO:0001960) | 3/24 | 55/14937 | 0.0000905 | 0.0018103 | 0.0011433 | *ISG15/OAS3/OAS1* | 3 |
| innate immune response (GO:0045087) | 5/24 | 302/14937 | 0.0001013 | 0.0018994 | 0.0011996 | *LTF/ISG15/OAS1/MX1/IFIT1* | 5 |
| regulation of monocyte chemotactic protein-1 production (GO:0071637) | 2/24 | 11/14937 | 0.0001349 | 0.0023803 | 0.0015034 | *OAS3/OAS1* | 2 |
| regulation of RNA metabolic process (GO:0051252) | 3/24 | 65/14937 | 0.0001491 | 0.0024854 | 0.0015697 | *IGF2BP2/OAS3/OAS1* | 3 |
| defense response to bacterium (GO:0042742) | 4/24 | 176/14937 | 0.0001646 | 0.0025993 | 0.0016417 | *LTF/ISG15/OAS3/OAS1* | 4 |
| regulation of chemokine (C-X-C motif) ligand 2 production (GO:2000341) | 2/24 | 13/14937 | 0.0001909 | 0.0028638 | 0.0018087 | *OAS3/OAS1* | 2 |
| positive regulation of type I interferon production (GO:0032481) | 3/24 | 77/14937 | 0.0002466 | 0.0034977 | 0.0022091 | *ISG15/OAS3/OAS1* | 3 |
| interleukin-27-mediated signaling pathway (GO:0070106) | 2/24 | 15/14937 | 0.0002565 | 0.0034977 | 0.0022091 | *OAS1/MX1* | 2 |
| negative regulation of ATPase activity (GO:0032780) | 2/24 | 17/14937 | 0.0003316 | 0.0041447 | 0.0026177 | *LTF/IFIT1* | 2 |
| negative regulation of chemokine production (GO:0032682) | 2/24 | 17/14937 | 0.0003316 | 0.0041447 | 0.0026177 | *OAS3/OAS1* | 2 |
| positive regulation of toll-like receptor signaling pathway (GO:0034123) | 2/24 | 27/14937 | 0.0008474 | 0.0101688 | 0.0064224 | *LTF/RSAD2* | 2 |
| regulation of tumor necrosis factor production (GO:0032680) | 3/24 | 124/14937 | 0.0009950 | 0.0114803 | 0.0072507 | *LTF/OAS3/OAS1* | 3 |
| positive regulation of bone mineralization (GO:0030501) | 2/24 | 36/14937 | 0.0015076 | 0.0167513 | 0.0105798 | *LTF/ISG15* | 2 |
| positive regulation of osteoblast differentiation (GO:0045669) | 2/24 | 44/14937 | 0.0022461 | 0.0240659 | 0.0151995 | *LTF/TP63* | 2 |
| iron ion transport (GO:0006826) | 2/24 | 50/14937 | 0.0028916 | 0.0299126 | 0.0188922 | *LTF/ATP6V0A4* | 2 |
| positive regulation of chemokine production (GO:0032722) | 2/24 | 53/14937 | 0.0032432 | 0.0324316 | 0.0204831 | *OAS3/OAS1* | 2 |
