# Supplementary material for: Low-grade glioma risk SNP rs11706832 is associated with type I interferon response pathway genes in cell lines
Source: Sci Rep. 2023 Apr 25;13:6777. doi: 10.1038/s41598-023-33923-4 (PMC10130147; doi:10.1038/s41598-023-33923-4)
Supplement: Supplementary file 14 — Supplementary Table S8. [file 41598_2023_33923_MOESM14_ESM.docx]

# S8. Differential Expression of genes in GO term GO:0071357 in TCGA LGG tumor samples. Conditioned on genotype at SNP position.

**baseMean**

mean normalized count across all samples

**log2FoldChange**

log_2_ fold change

**lfcSE**

standard error of log_2_ fold change

**pvalue**

p-value from Likelihood-ratio test

**padj**

Benjamini-Hochberg corrected p-value

|  | baseMean | log2FoldChange | lfcSE | stat | pvalue | padj | gene_name | chrom | start | end |
| --- | --- | --- | --- | --- | --- | --- | --- | --- | --- | --- |
| ENSG00000187608 | 2612,1569 | -0,2101482 | 0,2680343 | 27,9101882 | 0,0000009 | 0,000029 | *ISG15* | chr1 | 1001138 | 1014540 |
| ENSG00000111331 | 1880,7498 | -0,8213639 | 0,1854785 | 27,4134031 | 0,0000011 | 0,000029 | *OAS3* | chr12 | 112938444 | 112973251 |
| ENSG00000089127 | 813,99163 | -0,4252016 | 0,2018688 | 21,571275 | 0,0000207 | 0,0003587 | *OAS1* | chr12 | 112906783 | 112933222 |
| ENSG00000134321 | 556,81153 | -0,6262312 | 0,1889356 | 20,9735292 | 0,0000279 | 0,0003627 | *RSAD2* | chr2 | 6865806 | 6898239 |
| ENSG00000157601 | 1911,91714 | -0,5842342 | 0,1633353 | 19,5374635 | 0,0000572 | 0,000595 | *MX1* | chr21 | 41420304 | 41459214 |
| ENSG00000185745 | 4000,2495 | -0,3091364 | 0,1779097 | 18,6227105 | 0,0000904 | 0,0007834 | *IFIT1* | chr10 | 89392546 | 89406487 |
| ENSG00000185507 | 665,95163 | -0,420219 | 0,1679565 | 16,4964785 | 0,0002617 | 0,0019442 | *IRF7* | chr11 | 612553 | 615983 |
| ENSG00000126709 | 6611,38468 | -0,1830233 | 0,2360149 | 15,9571986 | 0,0003427 | 0,0022277 | *IFI6* | chr1 | 27666064 | 27672198 |
| ENSG00000117595 | 122,599 | 0,6420775 | 0,2003647 | 15,6731638 | 0,000395 | 0,0022823 | *IRF6* | chr1 | 209785617 | 209806175 |
| ENSG00000068079 | 1331,08984 | -0,2854518 | 0,1487144 | 15,2912973 | 0,0004781 | 0,0024862 | *IFI35* | chr17 | 43006740 | 43014456 |
| ENSG00000119917 | 4863,99938 | -0,3256537 | 0,1643908 | 11,9726015 | 0,0025129 | 0,0118794 | *IFIT3* | chr10 | 89327997 | 89340971 |
| ENSG00000213928 | 440,90679 | -0,3456162 | 0,1315595 | 10,6009873 | 0,0049891 | 0,0216196 | *IRF9* | chr14 | 24161265 | 24166565 |
| ENSG00000130303 | 1960,63994 | -0,0185641 | 0,165276 | 9,4322295 | 0,0089499 | 0,0357995 | *BST2* | chr19 | 17402939 | 17405630 |
| ENSG00000101347 | 6293,66385 | 0,2518136 | 0,1356317 | 8,2638459 | 0,016052 | 0,0596216 | *SAMHD1* | chr20 | 36890229 | 36951843 |
| ENSG00000204264 | 2068,43047 | -0,1727498 | 0,1283951 | 7,6939179 | 0,0213445 | 0,0704294 | *PSMB8* | chr6 | 32840717 | 32844679 |
| ENSG00000115415 | 6909,16208 | -0,2783555 | 0,1440801 | 7,6635987 | 0,0216706 | 0,0704294 | *STAT1* | chr2 | 190908460 | 191020960 |
| ENSG00000206503 | 24784,4187 | -0,1349823 | 0,129072 | 6,921242 | 0,0314103 | 0,0960784 | *HLA-A* | chr6 | 29941260 | 29945884 |
| ENSG00000125347 | 885,68226 | -0,3736604 | 0,1536434 | 6,302756 | 0,0427931 | 0,1236246 | *IRF1* | chr5 | 132481609 | 132490777 |
| ENSG00000135114 | 132,08955 | -0,0189433 | 0,2421535 | 5,9422309 | 0,0512461 | 0,1402525 | *OASL* | chr12 | 121019111 | 121039242 |
| ENSG00000204525 | 22766,0978 | -0,2240167 | 0,1373466 | 5,6570718 | 0,0590993 | 0,1536582 | *HLA-C* | chr6 | 31268749 | 31272130 |
| ENSG00000204592 | 23128,0284 | -0,0430989 | 0,1209692 | 5,2406931 | 0,0727776 | 0,1802113 | *HLA-E* | chr6 | 30489509 | 30494194 |
| ENSG00000204642 | 1189,76398 | -0,1277962 | 0,143594 | 5,1011352 | 0,0780374 | 0,1844519 | *HLA-F* | chr6 | 29722775 | 29738528 |
| ENSG00000126456 | 1661,19835 | 0,0050994 | 0,100936 | 4,8482073 | 0,0885575 | 0,2002169 | *IRF3* | chr19 | 49659569 | 49665875 |
| ENSG00000234745 | 28312,1554 | -0,1233602 | 0,1564846 | 4,685986 | 0,0960398 | 0,2010368 | *HLA-B* | chr6 | 31269491 | 31357188 |
| ENSG00000183486 | 289,7279 | -0,2332671 | 0,2184645 | 4,5859994 | 0,1009631 | 0,2010368 | *MX2* | chr21 | 41361999 | 41409393 |
| ENSG00000119922 | 3128,86305 | -0,2462174 | 0,1310243 | 4,5617629 | 0,1021941 | 0,2010368 | *IFIT2* | chr10 | 89283694 | 89309271 |
| ENSG00000111335 | 769,39343 | -0,3886223 | 0,1875405 | 4,519348 | 0,1043845 | 0,2010368 | *OAS2* | chr12 | 112978395 | 113011723 |
| ENSG00000142089 | 5086,70133 | 0,0635034 | 0,1523561 | 4,0602257 | 0,1313207 | 0,2431894 | *IFITM3* | chr11 | 319676 | 327537 |
| ENSG00000132530 | 212,94864 | -0,2811011 | 0,1643245 | 3,9957256 | 0,1356248 | 0,2431894 | *XAF1* | chr17 | 6755447 | 6775647 |
| ENSG00000185201 | 1231,8552 | 0,1155594 | 0,1382799 | 3,3435731 | 0,1879111 | 0,3174711 | *IFITM2* | chr11 | 307631 | 315272 |
| ENSG00000162645 | 2382,09469 | -0,2600023 | 0,2454562 | 3,3292502 | 0,1892616 | 0,3174711 | *GBP2* | chr1 | 89106132 | 89150456 |
| ENSG00000128604 | 708,37555 | -0,1674395 | 0,1505261 | 2,9661726 | 0,2269362 | 0,3592403 | *IRF5* | chr7 | 128937457 | 128950038 |
| ENSG00000168310 | 1710,30932 | 0,0509248 | 0,0682104 | 2,8673174 | 0,238435 | 0,3592403 | *IRF2* | chr4 | 184387729 | 184474550 |
| ENSG00000160710 | 19555,0779 | -0,0560773 | 0,0632131 | 2,8400874 | 0,2417035 | 0,3592403 | *ADAR* | chr1 | 154582057 | 154628013 |
| ENSG00000165949 | 1919,76453 | 0,0859695 | 0,1343605 | 2,8393188 | 0,2417964 | 0,3592403 | *IFI27* | chr14 | 94104836 | 94116698 |
| ENSG00000105397 | 4668,93197 | -0,0892467 | 0,0543405 | 2,7265305 | 0,2558241 | 0,3695237 | *TYK2* | chr19 | 10350529 | 10380572 |
| ENSG00000162434 | 11176,3315 | 0,0130753 | 0,096858 | 2,5379648 | 0,2811175 | 0,3870489 | *JAK1* | chr1 | 64833229 | 65067754 |
| ENSG00000184216 | 6689,70962 | -0,0423094 | 0,0665832 | 2,5257237 | 0,2828434 | 0,3870489 | *IRAK1* | chrX | 154010506 | 154019902 |
| ENSG00000067066 | 725,17781 | -0,1693981 | 0,1400253 | 2,4452996 | 0,2944489 | 0,3925985 | *SP100* | chr2 | 230415942 | 230545606 |
| ENSG00000185885 | 1510,26482 | 0,1310545 | 0,1936742 | 2,3418622 | 0,3100781 | 0,4031015 | *IFITM1* | chr11 | 313506 | 315272 |
| ENSG00000170581 | 4380,6916 | -0,1164197 | 0,0825582 | 2,1185953 | 0,3466992 | 0,4397161 | *STAT2* | chr12 | 56341597 | 56360167 |
| ENSG00000159110 | 918,25638 | -0,0614605 | 0,0613651 | 1,7656216 | 0,4136187 | 0,5120993 | *IFNAR2* | chr21 | 33229901 | 33265675 |
| ENSG00000120738 | 10802,3165 | 0,0429705 | 0,257332 | 1,66386 | 0,4352085 | 0,5262987 | *EGR1* | chr5 | 138465479 | 138469303 |
| ENSG00000172183 | 156,057 | -0,231211 | 0,1856804 | 1,5774665 | 0,4544201 | 0,5370419 | *ISG20* | chr15 | 88636153 | 88656483 |
| ENSG00000137265 | 40,95885 | 0,2113368 | 0,1979908 | 1,4901186 | 0,4747061 | 0,5485493 | *IRF4* | chr6 | 391752 | 411443 |
| ENSG00000140968 | 1087,71915 | 0,1220608 | 0,170471 | 1,4147559 | 0,492935 | 0,5572309 | *IRF8* | chr16 | 85899162 | 85922606 |
| ENSG00000068745 | 4462,71733 | -0,0306112 | 0,0496241 | 0,95712 | 0,6196751 | 0,685598 | *IP6K2* | chr3 | 48688003 | 48740353 |
| ENSG00000204632 | 92,36103 | -0,1566653 | 0,2008421 | 0,8420955 | 0,6563588 | 0,7110553 | *HLA-G* | chr6 | 29826967 | 29831125 |
| ENSG00000172936 | 1072,88821 | -0,1152813 | 0,1334764 | 0,7521725 | 0,6865431 | 0,7285764 | *MYD88* | chr3 | 38138478 | 38143022 |
| ENSG00000010704 | 143,41652 | 0,0610082 | 0,1123079 | 0,7082966 | 0,7017709 | 0,7298417 | *HFE* | chr6 | 26087281 | 26098343 |
| ENSG00000142166 | 5052,3699 | 0,0219335 | 0,0590433 | 0,3941905 | 0,8211124 | 0,8372126 | *IFNAR1* | chr21 | 33324429 | 33359864 |
| ENSG00000135828 | 646,10251 | -0,0171567 | 0,0862517 | 0,296393 | 0,8622617 | 0,8622617 | *RNASEL* | chr1 | 182573634 | 182589256 |
