# Supplementary material for: Low-grade glioma risk SNP rs11706832 is associated with type I interferon response pathway genes in cell lines
Source: Sci Rep. 2023 Apr 25;13:6777. doi: 10.1038/s41598-023-33923-4 (PMC10130147; doi:10.1038/s41598-023-33923-4)
Supplement: Supplementary file 15 — Supplementary Table S9. [file 41598_2023_33923_MOESM15_ESM.docx]

# S9. Concentration of metabolites in cell lines. Conditioned on allele on SNP position. A vs C

**p_val**

p-value from two sided t-test aggregating cell line replicates

**p_adj**

Benjamini-Hochberg adjusted **p_val**

**p_val_all**

p-value from two sided t-test not aggregating replicates

**p_adj_all**

Benjamini-Hochberg adjusted **p_val_all**

Table is sorted on **p_val_all**

| metabolite | p_val | p_val_all | p_adj | p_adj_all | log2_fc |
| --- | --- | --- | --- | --- | --- |
| ND RI:2058 | 0.0044076 | 0.0000479 | 0.2446220 | 0.0053206 | 0.6543918 |
| 3-Amino-2-piperidone | 0.0040801 | 0.0001016 | 0.2446220 | 0.0056404 | 0.5585617 |
| myo-Inositol | 0.0565437 | 0.0004116 | 0.3944882 | 0.0123342 | 0.2161958 |
| 2-Aminoadipic acid | 0.0842742 | 0.0004445 | 0.3944882 | 0.0123342 | 0.9909184 |
| O-Phosphoetanolamine | 0.0407987 | 0.0007815 | 0.3944882 | 0.0173494 | 0.3212991 |
| Hypotaurine | 0.1430595 | 0.0011880 | 0.4202819 | 0.0197004 | 0.5945773 |
| ND RI:1538 | 0.0514531 | 0.0012424 | 0.3944882 | 0.0197004 | 0.5866592 |
| Hexadecanoic acid, methyl ester | 0.0172578 | 0.0018918 | 0.3944882 | 0.0220596 | 0.3586356 |
| Sphingosine | 0.0765396 | 0.0019486 | 0.3944882 | 0.0220596 | 0.6731856 |
| ND RI:2590 | 0.0733934 | 0.0019874 | 0.3944882 | 0.0220596 | 0.4005124 |
| 6-Carbon Sugar Alcohol Mix 2 * | 0.0423868 | 0.0026441 | 0.3944882 | 0.0266811 | 0.3234762 |
| ND RI:2318 | 0.0683309 | 0.0032296 | 0.3944882 | 0.0283410 | 0.7492960 |
| scyllo-Inositol | 0.0924026 | 0.0033192 | 0.3944882 | 0.0283410 | 0.3581119 |
| Lysine | 0.0590192 | 0.0039515 | 0.3944882 | 0.0305262 | 0.3274602 |
| Pantothenic acid | 0.0562844 | 0.0041252 | 0.3944882 | 0.0305262 | -0.4750215 |
| Nicotinamide | 0.0868107 | 0.0046343 | 0.3944882 | 0.0316078 | 0.3273174 |
| Proline | 0.0996590 | 0.0048408 | 0.4097091 | 0.0316078 | -0.5368007 |
| Cystathionine | 0.1716801 | 0.0083842 | 0.4537261 | 0.0502015 | 0.4653608 |
| Lactose (Major) | 0.1083888 | 0.0085930 | 0.4202819 | 0.0502015 | 0.6182978 |
| Lathosterol | 0.1961979 | 0.0099482 | 0.4734340 | 0.0552124 | -0.5840565 |
| Histidine | 0.1411774 | 0.0140738 | 0.4202819 | 0.0734533 | 0.3803664 |
| Monomethylphosphate | 0.0711966 | 0.0145583 | 0.3944882 | 0.0734533 | 0.3389292 |
| ND RI:2103 | 0.1404474 | 0.0155459 | 0.4202819 | 0.0739103 | 0.3347101 |
| Glycerol-2-Phosphate | 0.1538718 | 0.0166285 | 0.4202819 | 0.0739103 | 0.5171347 |
| myo-Inositol-1-phosphate | 0.0591645 | 0.0166465 | 0.3944882 | 0.0739103 | 0.2526288 |
| Cholesterol | 0.1166266 | 0.0183296 | 0.4202819 | 0.0782534 | 0.2238113 |
| 1-Palmitoyl-Sn-Glycero-3-Phosphocholine | 0.0850292 | 0.0328121 | 0.3944882 | 0.1348942 | 0.2789871 |
| Urea | 0.0424539 | 0.0545614 | 0.3944882 | 0.2090697 | 0.2761510 |
| Serine | 0.1500448 | 0.0583900 | 0.4202819 | 0.2090697 | 0.4237409 |
| Adenosine-5-Monophosphate | 0.2501468 | 0.0597739 | 0.5339672 | 0.2090697 | 0.2414835 |
| 5-Carbon Sugar Alcohol Mix2 (Arabitol, Ribitol)* | 0.0878214 | 0.0624422 | 0.3944882 | 0.2090697 | -0.2332380 |
| Pyruvic acid | 0.1393390 | 0.0641626 | 0.4202819 | 0.2090697 | 0.1135805 |
| Adenosine | 0.0223064 | 0.0650441 | 0.3944882 | 0.2090697 | 0.3182341 |
| Glutamic acid | 0.0898674 | 0.0677279 | 0.3944882 | 0.2090697 | 0.1444760 |
| Orotic acid | 0.2311955 | 0.0686017 | 0.5339672 | 0.2090697 | -0.2954529 |
| Inosine | 0.0574125 | 0.0690644 | 0.3944882 | 0.2090697 | 0.3862979 |
| Dihydrouracil | 0.1876581 | 0.0708932 | 0.4687736 | 0.2090697 | 0.0899005 |
| Pyroglutamic acid | 0.0630833 | 0.0715734 | 0.3944882 | 0.2090697 | 0.1498293 |
| ND RI:1190 | 0.0545592 | 0.0737168 | 0.3944882 | 0.2098092 | 0.1972066 |
| Citrulline | 0.1900434 | 0.0790889 | 0.4687736 | 0.2194718 | 0.2274218 |
| Octadecenoic Acid,-9-(Z) | 0.1552392 | 0.0984462 | 0.4202819 | 0.2577222 | 0.1591033 |
| Isoleucine | 0.0694059 | 0.0988164 | 0.3944882 | 0.2577222 | 0.4365689 |
| Uridine | 0.2717152 | 0.1011796 | 0.5385784 | 0.2577222 | 0.6074859 |
| Pyrazine | 0.1218185 | 0.1029951 | 0.4202819 | 0.2577222 | 0.3015197 |
| Citric acid | 0.4044758 | 0.1053317 | 0.6802548 | 0.2577222 | 0.2487886 |
| Ketohexose Mix 1 (Fructose, Sorbose, Tagatose, Psicose)* | 0.4411775 | 0.1068038 | 0.7302227 | 0.2577222 | 0.4516782 |
| Aldohexose Mix 1 (Galactose/Gulose/Allose)* | 0.1361199 | 0.1134860 | 0.4202819 | 0.2680201 | -0.2254470 |
| Aminomalonic acid | 0.3697982 | 0.1315651 | 0.6620580 | 0.3042443 | 0.1501402 |
| 1,6-Anhydro-Beta-D-Glucose | 0.1367521 | 0.1387073 | 0.4202819 | 0.3069220 | 0.1407636 |
| Fructose-6-Phosphate | 0.2587325 | 0.1407286 | 0.5351679 | 0.3069220 | 0.3693472 |
| Tyrosine | 0.3443059 | 0.1424882 | 0.6280523 | 0.3069220 | 0.1966672 |
| Glucose-6-Phosphate | 0.3333795 | 0.1437833 | 0.6272054 | 0.3069220 | 0.3778701 |
| Lactose (Minor) | 0.2773327 | 0.1526651 | 0.5400689 | 0.3105515 | 0.2970291 |
| Alanine | 0.1860760 | 0.1531409 | 0.4687736 | 0.3105515 | 0.3095478 |
| Leucine | 0.3112906 | 0.1538769 | 0.5957458 | 0.3105515 | 0.0777157 |
| Adenine | 0.1436230 | 0.1726971 | 0.4202819 | 0.3423103 | -0.1131390 |
| Octadecanoic acid | 0.2007796 | 0.1764512 | 0.4741815 | 0.3436156 | 0.1354232 |
| ND RI:1577 | 0.1458893 | 0.1857264 | 0.4202819 | 0.3554419 | -0.1518657 |
| 2-Hydroxyglutaric acid | 0.2712641 | 0.2084813 | 0.5385784 | 0.3922276 | -0.0514865 |
| 1-Aminocyclopropane carboxylic acid | 0.4510773 | 0.2138354 | 0.7302227 | 0.3955955 | -0.3859697 |
| Aspartic acid | 0.5255627 | 0.2261922 | 0.7883440 | 0.4093807 | 0.2631801 |
| ND RI:1167 | 0.3893503 | 0.2286631 | 0.6725180 | 0.4093807 | 0.2076222 |
| Tryptophan | 0.2464445 | 0.2921089 | 0.5339672 | 0.5146681 | 0.1592372 |
| Maltose | 0.3834060 | 0.3032541 | 0.6725180 | 0.5259563 | -0.1806596 |
| Lactic acid | 0.4539222 | 0.3117269 | 0.7302227 | 0.5323337 | 0.2971948 |
| Aldopentose Mix (Xylose, Lyxose)* | 0.2603519 | 0.3235025 | 0.5351679 | 0.5440723 | 0.1014207 |
| Taurine | 0.2498534 | 0.3333105 | 0.5339672 | 0.5522010 | 0.4764727 |
| Cysteine | 0.4882001 | 0.3413863 | 0.7526418 | 0.5572629 | 0.1734647 |
| Threonine | 0.4618360 | 0.3786583 | 0.7302251 | 0.6091460 | 0.1717058 |
| Aldohexose Mix 2 (Glucose, Galactose, Mannose, Idose, Altrose)* | 0.4670809 | 0.3899045 | 0.7302251 | 0.6182771 | 0.1008541 |
| Glucose-1-Phosphate | 0.3451459 | 0.4104860 | 0.6280523 | 0.6386490 | 0.1245300 |
| Fucose | 0.2478798 | 0.4142588 | 0.5339672 | 0.6386490 | 0.1054776 |
| Threitol | 0.3938169 | 0.4334954 | 0.6725180 | 0.6591506 | 0.0842499 |
| Aldohexose Mix 3 (Glucose, Galactose, Mannose, Altrose, Talose, Gulose)* | 0.7040406 | 0.4412075 | 0.8704502 | 0.6618113 | 0.0391773 |
| Beta-Alanine | 0.6927409 | 0.4578869 | 0.8704502 | 0.6762583 | -0.1069600 |
| Ketohexose Mix 2 (Fructose, Sorbose, Tagatose, Psicose)* | 0.6913190 | 0.4659734 | 0.8704502 | 0.6762583 | 0.2052024 |
| N-acetyl-L-serine | 0.6830580 | 0.4691161 | 0.8704502 | 0.6762583 | -0.0920995 |
| Gamma-aminobutyric acid (GABA) | 0.6111950 | 0.4863663 | 0.8697775 | 0.6903742 | 0.1197114 |
| 6-Carbon Sugar Alcohol Mix 1 (Mannitol, Sorbitol, Galactitol, Iditol)* | 0.7546020 | 0.4984105 | 0.8704502 | 0.6903742 | 0.2905419 |
| Asparagine | 0.6302013 | 0.5049145 | 0.8704502 | 0.6903742 | 0.1154623 |
| Cysteine-Glycine | 0.4961854 | 0.5078013 | 0.7544737 | 0.6903742 | 0.1198767 |
| Gluconic acid | 0.7386573 | 0.5158931 | 0.8704502 | 0.6903742 | 0.1193987 |
| Spermidine | 0.6623298 | 0.5316545 | 0.8704502 | 0.6903742 | 0.0904364 |
| Palmitoleic acid | 0.6771299 | 0.5335387 | 0.8704502 | 0.6903742 | 0.0588332 |
| Creatinine | 0.5728783 | 0.5341532 | 0.8478599 | 0.6903742 | 0.1088252 |
| Glutamine | 0.6895466 | 0.5348845 | 0.8704502 | 0.6903742 | 0.4282486 |
| 1-Monostearoylglycerol | 0.5825868 | 0.5725552 | 0.8508834 | 0.7198417 | 0.1183891 |
| Valine | 0.6005775 | 0.5729225 | 0.8657675 | 0.7198417 | 0.0821881 |
| Cytidine-5'-monophosphate | 0.7305822 | 0.5771704 | 0.8704502 | 0.7198417 | 0.1051400 |
| Argininosuccinate | 0.7058396 | 0.6324436 | 0.8704502 | 0.7800137 | 0.1237441 |
| Glycylglycine | 0.6591457 | 0.6408699 | 0.8704502 | 0.7817204 | 0.0572760 |
| Methionine | 0.7852568 | 0.6514203 | 0.8793040 | 0.7859527 | -0.0520376 |
| Uridine 5'-Diphospho-N-Acetylglucosamine | 0.8088905 | 0.6642463 | 0.8793040 | 0.7918383 | 0.0622347 |
| Guanosine | 0.7192853 | 0.6713038 | 0.8704502 | 0.7918383 | -0.1277954 |
| Fructose-1-Phosphate | 0.8188036 | 0.6778635 | 0.8793040 | 0.7918383 | -0.1246584 |
| ND RI:2262 | 0.8238524 | 0.6893747 | 0.8793040 | 0.7918383 | 0.0723991 |
| Phenylalanine | 0.7606637 | 0.6934179 | 0.8704502 | 0.7918383 | 0.0331286 |
| S-adenosyl-L-methionine (SAM) | 0.6285394 | 0.6991005 | 0.8704502 | 0.7918383 | 0.0433298 |
| Sucrose | 0.7327576 | 0.7408194 | 0.8704502 | 0.8306156 | 0.0717642 |
| Ornithine | 0.7686500 | 0.7567277 | 0.8706138 | 0.8399677 | -0.0610815 |
| Xylulose | 0.8211396 | 0.7706901 | 0.8793040 | 0.8469960 | -0.0357422 |
| Asparagine [-H2O] | 0.7539432 | 0.7823498 | 0.8704502 | 0.8513807 | 0.0407807 |
| Succinic acid | 0.7567020 | 0.8007322 | 0.8704502 | 0.8629250 | 0.0525480 |
| Hypoxanthine | 0.8173648 | 0.8145017 | 0.8793040 | 0.8693239 | 0.0662657 |
| Uracil | 0.8998056 | 0.8509517 | 0.9339047 | 0.8932934 | 0.0214206 |
| 2-Methylmalic acid | 0.8926009 | 0.8552531 | 0.9339047 | 0.8932934 | -0.0236230 |
| Glycine | 0.9138996 | 0.8611027 | 0.9392857 | 0.8932934 | 0.0286672 |
| Fumaric acid | 0.9002505 | 0.9038053 | 0.9339047 | 0.9289110 | 0.0093783 |
| 5-Carbon Sugar Alcohol Mix 1 (Xylitol, Ribitol)* | 0.9446593 | 0.9233446 | 0.9619925 | 0.9344209 | -0.0144799 |
| N-acetyl-L-aspartic acid | 0.9583188 | 0.9260027 | 0.9670308 | 0.9344209 | 0.0151652 |
| Glycerol-3-Phosphate | 0.9822345 | 0.9674013 | 0.9822345 | 0.9674013 | 0.0071179 |
