# Supplementary material for: Low-grade glioma risk SNP rs11706832 is associated with type I interferon response pathway genes in cell lines
Source: Sci Rep. 2023 Apr 25;13:6777. doi: 10.1038/s41598-023-33923-4 (PMC10130147; doi:10.1038/s41598-023-33923-4)
Supplement: Supplementary file 16 — Supplementary Table S10. [file 41598_2023_33923_MOESM16_ESM.docx]

# S10. Principal components on 111 metabolites from cell lines.

|  | Standard.deviation | Proportion.of.Variance | Cumulative.Proportion | t_pval_pca |
| --- | --- | --- | --- | --- |
| PC1 | 6,3703423 | 0,3656 | 0,3656 | 0,0727813 |
| PC2 | 3,8591148 | 0,13417 | 0,49977 | 0,7703583 |
| PC3 | 3,3405118 | 0,10053 | 0,6003 | 0,0898091 |
| PC4 | 2,7484505 | 0,06805 | 0,66835 | 0,6334074 |
| PC5 | 2,2331569 | 0,04493 | 0,71328 | 0,4325451 |
| PC6 | 2,0922199 | 0,03944 | 0,75272 | 0,1145866 |
| PC7 | 1,7002592 | 0,02604 | 0,77876 | 0,2698805 |
| PC8 | 1,586312 | 0,02267 | 0,80143 | 0,6439812 |
| PC9 | 1,5275758 | 0,02102 | 0,82245 | 0,6091051 |
| PC10 | 1,4407095 | 0,0187 | 0,84115 | 0,1221581 |
| PC11 | 1,3349615 | 0,01606 | 0,85721 | 0,4808594 |
| PC12 | 1,2844779 | 0,01486 | 0,87207 | 0,7356917 |
| PC13 | 1,227456 | 0,01357 | 0,88564 | 0,3684816 |
| PC14 | 1,149186 | 0,0119 | 0,89754 | 0,7275633 |
| PC15 | 1,0973658 | 0,01085 | 0,90839 | 0,7229206 |
| PC16 | 1,0733049 | 0,01038 | 0,91877 | 0,78308 |
| PC17 | 1,0557154 | 0,01004 | 0,92881 | 0,6160949 |
| PC18 | 1,0212944 | 0,0094 | 0,93821 | 0,8531691 |
| PC19 | 0,9356564 | 0,00789 | 0,94609 | 0,2256908 |
| PC20 | 0,913728 | 0,00752 | 0,95361 | 0,6447572 |
| PC21 | 0,8460846 | 0,00645 | 0,96006 | 0,5156752 |
| PC22 | 0,7867657 | 0,00558 | 0,96564 | 0,9185888 |
| PC23 | 0,7090923 | 0,00453 | 0,97017 | 0,4082412 |
| PC24 | 0,6582688 | 0,0039 | 0,97407 | 0,6575066 |
| PC25 | 0,651005 | 0,00382 | 0,97789 | 0,1798383 |
| PC26 | 0,617003 | 0,00343 | 0,98132 | 0,3308058 |
| PC27 | 0,5763253 | 0,00299 | 0,98431 | 0,2394345 |
| PC28 | 0,5363736 | 0,00259 | 0,98691 | 0,8054128 |
| PC29 | 0,5240675 | 0,00247 | 0,98938 | 0,1151728 |
| PC30 | 0,4995601 | 0,00225 | 0,99163 | 0,8309275 |
| PC31 | 0,4860511 | 0,00213 | 0,99376 | 0,894484 |
| PC32 | 0,4490921 | 0,00182 | 0,99557 | 0,4134415 |
| PC33 | 0,4024398 | 0,00146 | 0,99703 | 0,9842245 |
| PC34 | 0,3641134 | 0,00119 | 0,99823 | 0,3530753 |
| PC35 | 0,322974 | 0,00094 | 0,99917 | 0,0936239 |
| PC36 | 0,3040907 | 0,00083 | 1 | 0,2972811 |
| PC37 | 0 | 0 | 1 | 0,5612502 |
